# Marine-Derived Chitooligosaccharide Attenuates Obesity and Metabolic Syndrome in Bama Pigs Through LXR-Mediated Cholesterol Metabolism and Gut Microbiota Modulation

**DOI:** 10.3390/nu18081233

**Published:** 2026-04-14

**Authors:** Minchuan Zhou, Kaiwen Lei, Jiahua Zhang, Qihao Yan, Hua Cao, Yan Bai, Kunhua Wei, Zhengquan Su

**Affiliations:** 1 Guangdong Engineering Research Center of Natural Products and New Drugs, Guangdong Provincial University Engineering Technology Research Center of Natural Products and Drugs, Guangdong Pharmaceutical University, Guangzhou 510006, China; zhouminchuan2021@163.com (M.Z.); 18272834470@163.com (K.L.); 13188236390@163.com (J.Z.); yanqihao980919@163.com (Q.Y.); 2 Clinical Medical Department, Chengdu University of Traditional Chinese Medicine, Chengdu 610072, China; 3 Guangdong Metabolic Disease Research Center of Integrated Chinese and Western Medicine, Key Laboratory of Glucolipid Metabolic Disorder, Ministry of Education of China, Institute of Chinese Medicine, Guangdong TCM Key Laboratory for Metabolic Diseases, Guangdong Pharmaceutical University, Guangzhou 510006, China; 4 School of Chemistry and Chemical Engineering, Guangdong Pharmaceutical University, Zhongshan 528458, China; caohua@gdpu.edu.cn; 5 School of Public Health, Guangdong Pharmaceutical University, Guangzhou 510310, China; angell_bai@163.com; 6 Key Laboratory of State Administration of Traditional Chinese Medicine for Production & Development of Cantonese Medicinal Materials, Guangzhou Comprehensive Experimental Station of National Industrial Technology System for Chinese Materia Medica, Guangdong Engineering Research Center of Good Agricultural Practice & Comprehensive Development for Cantonese Medicinal Materials, School of Chinese Materia Medica, Guangdong Pharmaceutical University, Guangzhou 510006, China

**Keywords:** obesity, metabolic syndrome, Bama pig, Chitooligosaccharides, cholesterol metabolism, gut microbiota-BA-SCFA network

## Abstract

**Background/Objectives:** Chitooligosaccharide (COS) is a marine-derived natural product obtained from shrimp and crab shells. Although its anti-inflammatory and antioxidant activities are documented, its potential effects on obesity and metabolic syndrome remain largely unclear. This study aimed to investigate the efficacy of COST (MW ≈ 1000 Da) against high-fat diet (HFD)-induced obesity and metabolic syndrome in Bama pigs. **Methods:** Bama pigs were fed a HFD for 12 weeks to establish an obesity model, followed by 12 weeks of oral COST administration. Serum biochemical parameters, tissue indicators, histopathology, and gene/protein expression related to cholesterol metabolism were analyzed. Fecal bile acid (BA) profiles, gut microbiota composition, and short-chain fatty acid (SCFA) levels were also examined. **Results:** COST treatment significantly attenuated weight gain and improved multiple components of metabolic syndrome, including insulin resistance, dyslipidemia, and inflammation. Mechanistically, COST upregulated intestinal ABCG5/ABCG8 to promote cholesterol excretion, increased ABCA1 expression in intestine and liver to enhance reverse cholesterol transport (RCT), and upregulated hepatic LDL-R to facilitate LDL-C clearance from circulation while modulating hepatic cholesterol synthesis via SREBP2 downregulation and RNF145 upregulation. These transcriptional changes were confirmed at the protein level for LXR, LDL-R, and ABCA1. Additionally, COST decreased fecal secondary BA levels, reshaped gut microbiota composition, and increased SCFA production, with significant correlations among these factors. **Conclusions:** COST ameliorates protective effects against HFD-induced obesity and metabolic syndrome, potentially through the regulation of cholesterol metabolism and the modulation of the gut microbiota-BA-SCFA network.

## 1. Introduction

Obesity is linked to a range of adverse health outcomes, including type 2 diabetes mellitus (T2DM), cardiovascular disease (CVD), chronic kidney disease (CKD), and certain cancers [1,2,3]. According to World Health Organization (WHO) data, the global prevalence of obesity among adults has more than doubled since 1990, while the rate among adolescents has tripled. Moreover, a World Obesity Federation (WOF) report warns that without urgent and effective interventions to contain this expanding epidemic, over 4 billion people, nearly half the world’s population, will be living with overweight/obesity by 2035 [4]. More critically, obesity is a major risk factor for a cluster of metabolic abnormalities collectively defined as metabolic syndrome, including central obesity, insulin resistance, dyslipidemia, and hypertension [5]. The global prevalence of metabolic syndrome has risen in parallel with the obesity epidemic, dramatically increasing the burden of T2DM and cardiovascular disease [6]. Therefore, identifying effective strategies that simultaneously target multiple components of metabolic syndrome is of paramount importance.

Lifestyle modifications alone are often challenging and insufficient for maintaining long-term weight loss. Therefore, pharmacotherapy should be considered in a timely manner for overweight or obese patients. Orlistat, the only FDA-approved pancreatic lipase inhibitor for long-term use, is widely prescribed as an anti-obesity medication. It exerts its therapeutic effects by inhibiting gastric and pancreatic lipases, thereby reducing dietary fat absorption. However, despite its therapeutic efficacy, reports indicate that 91% of patients receiving Orlistat treatment experience gastrointestinal disturbances and other adverse effects [7], underscoring the critical need for therapeutic alternatives with enhanced safety and superior tolerability.

In this context, COS, a marine-derived natural product obtained from shrimp and crab shells, has emerged as a promising dietary bioactive compound for metabolic health. The process of converting these shells into COS involves several key steps: collected shrimp and crab shells are first washed, dried, deproteinized, and decalcified sequentially to yield chitin. Chitin is then subjected to high-temperature treatment with concentrated alkali to undergo deacetylation, thereby converting into chitosan, a polysaccharide composed of glucosamine and *N*-acetylglucosamine linked by β-1,4 glycosidic bonds. Finally, chitosan is degraded into oligosaccharides with a polymerization degree of 2–10, which are COS [8]. In recent years, modern studies have extensively explored the biological activities of COS. Accumulating evidence indicates that COS exerts multiple beneficial effects, including enhancing immune function, regulating lipid metabolism, lowering blood glucose, and improving insulin sensitivity [9]. Given its comprehensive regulatory actions on glucose and lipid metabolism, COS exhibits considerable potential as a candidate for the prevention and treatment of obesity.

Animal models play an indispensable role in exploring the pathogenesis of human diseases and developing preventive and therapeutic strategies [10]. Rodents are currently the most commonly used preclinical models for studying metabolic disorders. Our previous studies have demonstrated that COS ameliorated obesity-related indicators, including body weight gain, fat pad lipid accumulation, and hepatic steatosis in obese SD rats and C57BL/6 mice [11]. However, rodents exhibit inherent physiological divergences from humans that critically limit direct clinical translation, particularly concerning lipid and bile acid metabolism [12]. Specifically, rodents are “HDL-predominant” mammals and naturally lack cholesteryl ester transfer protein (CETP), whereas humans and pigs are “LDL-predominant” and share highly similar lipoprotein metabolism profiles. Furthermore, the rodent bile acid pool is characterized by murine-specific muricholic acids, whereas the porcine bile acid composition and enterohepatic circulation closely mirror those of humans. Given that COST acts primarily in the gastrointestinal tract as an indigestible marine polysaccharide, the anatomical structure, transit time, and baseline microbiota composition of the porcine gut provide a far superior translational platform for evaluating gut–microbiome interactions [13]. Therefore, to bridge the crucial translational gap between preliminary rodent studies and human clinical applications, the present study employed a high-fat diet (HFD)-induced obese Bama mini-pig model. Rather than merely confirming the general anti-obesity effects previously observed in rodents, the main contribution of this study lies in comprehensively evaluating COST within this highly translatable porcine model while integrating macroscopic metabolic assessments, detailed bile acid profiling, and targeted gut microbiota analyses.

## 2. Materials and Methods

### 2.1. Chemicals and Reagents

COST (MW = 1060 Da; deacetylation degree > 90%; lot No. 211009C) was purchased from Shandong AK Biotech Co., Ltd. (Qingdao, China) under the NMPA approval number H20143119. To guarantee the biological safety of this intervention, rigorous quality control was ensured. The accompanying Certificate of Analysis (Table S1) confirmed that the COST extract was strictly sterile and devoid of any microbial contamination prior to the animal feeding trials. Furthermore, Orlistat, used as the positive control, was acquired from Zhongshan Wanhan Pharmaceutical Co., Ltd. (Guangzhou, China). The enzymatic determination of alanine aminotransferase (ALT), aspartate aminotransferase (AST), and fasting blood glucose (FBG) was conducted according to the manufacturer’s instructions (Nanjing Jiancheng Bioengineering Institute, Nanjing, China). Similarly, lipid-related markers, specifically total cholesterol (TC), triglycerides (TG), total bile acid (TBA), non-esterified fatty acids (NEFA), low-density lipoprotein cholesterol (LDL-C), and high-density lipoprotein cholesterol (HDL-C), were quantified using the respective colorimetric kits (Nanjing Jiancheng Bioengineering Institute, Nanjing, China). Enzyme-linked immunosorbent assay (ELISA) kits for the determination of insulin (INS, Cat. No. 0390), lipopolysaccharide (LPS, Cat. No. 0781), tumor necrosis factor-α (TNF-α, Cat. No. 0383), interleukin-1β (IL-1β, Cat. No. 0422), interleukin-6 (IL-6, Cat. No. 0418), and interleukin-10 (IL-10, Cat. No. 0425) were obtained from Jiangsu Meimian Industrial Co., Ltd. (Yancheng, China).

### 2.2. Animal Experimental Design and Sample Collection

Healthy Chinese Bama minipigs (Sus scrofa domestica), aged 5 months and weighing 20–30 kg, were obtained from Guangzhou Zhongke Flying Dolphin Biotechnology Co., Ltd. (Guangzhou, Guangdong, China). To ensure a highly consistent genetic background and minimize biological variance, fourteen near-siblings were specifically selected from two synchronized litters derived from the same maternal lineage. These animals comprised a neutered mixed-sex cohort; to eliminate the potential confounding effects of endogenous sex hormones on metabolic and inflammatory profiles, all female and male minipigs had undergone bilateral ovariectomy or surgical castration, respectively, at approximately one month of age (pre-pubertal stage). Upon arrival, each miniature pig was housed individually in a temperature-controlled compartment. Animal procedures were conducted in strict accordance with the Guide for the Care and Use of Laboratory Animals (National Institutes of Health). The experimental protocol received formal ethical clearance from the Institutional Animal Care and Use Committee (IACUC) of Guangdong Pharmaceutical University (Experiment No. GDPULAC2021286). Humane endpoints were established to prevent unnecessary suffering: animals showing severe symptoms, such as weight loss exceeding 20%, complete anorexia for 48 h, or clinical signs of systemic infection, would be euthanized immediately. No animals reached these predefined endpoints during this study.

**Figure 1:**
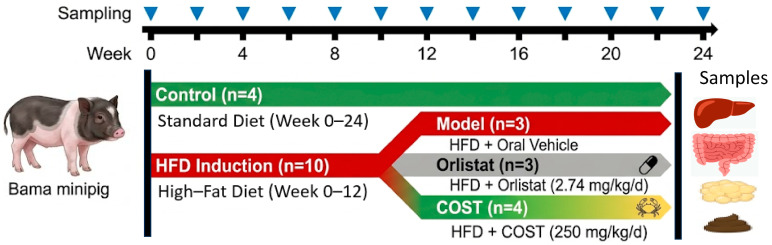
Overview of the experimental workflow and sample collection. Animal experiments are divided into an obesity induction phase (Week 0–12) and a treatment phase (Week 12–24), preceded by a 7-day adaptation period. Bama minipigs (*n* = 14) were initially divided into a Control group (*n* = 4, standard diet) and a high-fat feed group (HF group, *n* = 10) for 12 weeks. Following the induction phase, the HF group was randomly subdivided into a Model group (*n* = 3), Orlistat group (*n* = 3, 2.74 mg/kg/d), and COST group (*n* = 4, 250 mg/kg/d) for a 12-week oral intervention. Sampling time points for serum, feces, and physiological measurements are marked by blue triangles. Final terminal sampling at week 24 included blood, liver, ileum, colon, cecum contents, and adipose tissues.

After 7 days of adaptive feeding, fourteen near-siblings of the same age were allocated into a normal food group (Control group, *n* = 4) with a continuous standard diet, and high-fat feed group (HF group, *n* = 10) with HFD. As body weight is a critical parameter for establishing the obesity model, the animals were ranked by weight: individuals with slightly lower weights were assigned to the Control group to serve as a physiological reference, while the others were assigned to the HF group. No statistically significant difference in baseline weight was observed between the two cohorts (*p* > 0.05). The comprehensive experimental design and sampling timeline are illustrated in Figure 1. The standard diet contains 25.238% corn, 4% imported fishmeal, 8% soybean meal, 10% rice bran, 20% wheat bran, 30% alfalfa grass powder, 2.4% calcium bicarbonate, 0.3% iodized salt, 0.03% trace element additives, and 0.032% vitamin additives. The HFD contains 49.5% standard feed, 10% butter, 10% margarine, 15% sucrose, 10% casein, 2% experimental animal premix, 1.5% microcrystalline cellulose, and 2% calcium bicarbonate. The feed was customized in Chengdu Hualanxu Biotechnology Co., Ltd. (Chengdu, Sichuan, China). Following 12 weeks of HFD induction, minipigs in the HF group were further allocated into the Model group (*n* = 3), Orlistat group (*n* = 3), and COST group (*n* = 4) using stratified randomization based on their body weight at week 12. This secondary allocation ensured a uniform phenotypic baseline across the three intervention groups prior to treatment. The cohort scale (*n* = 3–4 per group) was determined following consultation with Professor Bei and was informed by validated porcine metabolic protocols previously established within our institution. As observed in the referenced minipig model of diabetes and coronary heart disease, a sample size of *n* = 3 provided sufficient biological resolution to resolve effect sizes in glucose and lipid metabolism, such as the marked elevations in serum TG and TC [14]. This approach is further supported by the minimized biological variance among the near-siblings selected and aligns with the ‘Reduction’ principle of the 3Rs for large experimental mammals, aiming to balance scientific integrity with ethical considerations.

Following the induction phase, the Orlistat group and COST group were given Orlistat (2.74 mg/kg/d) and COST (250 mg/kg/d), respectively, via oral administration. Orlistat served as a positive control [15]. These doses were determined based on our preliminary laboratory data and scaled using the standard body surface area conversion coefficient (Rab = 1.370). Meanwhile, the Control group continued on a standard diet, and the Model group remained on HFD without pharmacological intervention. The specific experimental diets and dosages are summarized in Table S2. To maintain the objectivity of this study and mitigate potential observer bias, a structured blinding protocol was implemented for pharmacological administration. Two weeks’ supply of individual doses for the Orlistat and COST groups was pre-weighed and sealed into light-shielding aluminum foil sachets by laboratory personnel who were not involved in daily animal husbandry. Each sachet was labeled exclusively with a corresponding animal identification number, concealing the specific treatment and dosage from the animal care staff. These coded aliquots were subsequently transferred to independent facility personnel for daily distribution. During administration, the staff incorporated the pre-measured contents into approximately one-third of the daily feed ration. The remaining diet was provided only after the medicated fraction had been entirely consumed, thereby ensuring consistent and complete dose compliance. Throughout the intervention period, the physiological status of all minipigs was monitored and documented daily by the blinded facility staff.

After 12 weeks of oral treatment, the minipigs were fasted for 16 h with water and then weighed. Simultaneously, the body dimension was measured. To ensure a painless and stress-free euthanasia, minipigs were first sedated with an intramuscular injection of midazolam (0.2 mg/kg), followed by deep anesthesia induced by isoflurane (4–5% for induction, 2–3% for maintenance via mask). Once a surgical plane of anesthesia was confirmed (loss of pedal reflex), blood from the heart chambers was quickly and gently collected. The animals were then humanely sacrificed via exsanguination under deep anesthesia. Next, the subcutaneous fat thickness of the back and abdomen was measured by vernier calipers. After being dissected, the whole heart, liver, spleen, kidney and the perirenal fat of Bama pigs were weighed. Last, these tissues, including liver, ileum, colon, perirenal fat (as vWAT), abdominal subcutaneous fat (as sWAT) and cecum contents, were collected and stored at −80 °C. Portions of them were fixed in 4% paraformaldehyde for histological analysis.

### 2.3. Measurement of Body Weight and Dimensions

Body weight was measured using a large-capacity loadometer. The obesity index, defined as [(actual weight of HF group—mean weight of Control group)/mean weight of Control group] × 100%, was calculated to assess the success of the obesity model and the efficacy of the intervention; an obesity index greater than 20% was considered indicative of obesity.

Body dimensions, including body length, neck circumference, chest circumference, abdominal circumference, and waist circumference, were measured using a tape measure. Body length was defined as the straight-line distance from the midpoint between the ears, along the backbone, to the base of the tail. Neck circumference was measured as the loop around the neck passing through the posterior edge of the ear. Chest and abdominal circumferences were measured by wrapping the tape around the body at the posterior edge of the shoulder blades and the abdomen, respectively. Waist circumference was measured at the base of the hind legs.

### 2.4. Biochemical Analysis

Serum biochemical analysis: After 30 min incubation at room temperature, blood samples were centrifuged (1500× *g*, 15 min, 4 °C) to isolate the serum. Concentrations of FBG, ALT, AST, and TBA, as well as a lipid profile including TG, TC, HDL-C, LDL-C, and NEFA, were determined using commercial enzymatic kits (Nanjing Jiancheng Bioengineering Institute, Nanjing, China). Additionally, levels of INS, LPS, and inflammatory cytokines such as IL-1β, IL-6, IL-10, and TNF-α were measured via ELISA according to the provided protocols. The homeostatic model assessment of insulin resistance (HOMA-IR) was calculated using the formula: HOMA-IR = FBG (mmol/L) × INS (mU/L)/22.5.

Tissue biochemical analysis: Briefly, 1.5 mL microcentrifuge tubes were prepared with 0.9 mL of physiological saline and two grinding beads to contain 100 mg of tissue samples. The mixture was processed using an automated homogenizer to ensure complete tissue disruption. Following 10 min centrifugation (600× *g* at 4 °C), the resulting supernatants were harvested for subsequent assays. Liver tissue levels of GLU, ALT, AST, TG, TC, and LDL-C were measured using commercial kits (Nanjing Jiancheng Bioengineering Institute, Nanjing, China). Additionally, inflammatory cytokines in adipose tissue (IL-1β, IL-6, IL-10, and TNF-α) were quantified via ELISA according to the provided protocols (Meimian Industrial, Yancheng, China).

Fecal biochemical analysis: Dry fecal samples (approximately 500 mg) were placed in centrifuge tubes containing two grinding beads, followed by the addition of 1.8 mL of ether. The tubes were shaken vigorously and then placed on a shaker for 15 min. After centrifugation, the supernatant was collected. Fecal TC and TG levels were measured using commercial kits from Nanjing Jiancheng Bioengineering Institute (Nanjing, China), with TG measurement performed according to previously established laboratory methods. All procedures were performed in strict accordance with the manufacturers’ instructions, and each sample was analyzed in triplicate.

### 2.5. Histopathological Analysis

The tissue samples of liver, fat, ileum and colon were fixed in 4% paraformaldehyde solution for 48 h. Next, they were, in turn, dehydrated through gradient alcohol, permeabilized by xylene, and embedded in paraffin wax. The embedded tissues were cut into about 6 µm thick sections, which were stained in hematoxylin for 5 min and eosin for 15 s (Leagene Biotechnology, Beijing, China). The stained sections were then stored for microscopic observation. To safeguard objectivity and mitigate detection bias, the semi-quantitative scoring of vWAT adipocytes and all subsequent image analyses were conducted by investigators blinded to the treatment groups. Samples were identified solely by unique numerical codes, and group identities remained concealed until the final statistical analysis was completed.

### 2.6. Quantitative RT-PCR Assay

Target gene sequences were identified via the NCBI database, and the corresponding primers were synthesized by Sangon Biotech (Shanghai, China), as detailed in Table S3. Total RNA was isolated from the liver, ileum, and colon tissues of the minipigs. We assessed the concentration and purity of the harvested RNA using a NanoDrop 2000 spectrophotometer. To eliminate potential genomic DNA interference, RNA templates underwent a 2 min incubation with gDNA Eraser at 42 °C prior to the reverse transcription process. Subsequently, 1 μL of total RNA was utilized for cDNA synthesis in a 20 μL reaction, following the protocol provided with the RR047 Reverse Transcription Kit (TaKaRa Bio, Shiga, Japan). The qPCR was performed on a Roche 96-well plate using the TB Green Premix Ex Taq II kit (TaKaRa). Each reaction mixture contained 2 μL of cDNA along with specific primers and DEPC-treated water. Thermal cycling was conducted on a LightCycler 480 system (Roche, Basel, Switzerland), starting with an initial denaturation at 95 °C for 30 s, followed by 40 cycles of 95 °C for 5 s and 60 °C for 30 s, and concluding with a 7 min extension at 72 °C. We calculated the relative mRNA expression levels using the 2^−∆∆Ct^ method, with β-actin serving as the internal control for normalization. Fold-change values for all intervention groups were determined relative to the Control group, which functioned as the experimental calibrator.

### 2.7. Western Blot (WB) Assay

Liver tissues were lysed using RIPA buffer supplemented with protease and phosphatase inhibitors (Meilunbio, Dalian, China). To eliminate insoluble debris, the homogenates underwent centrifugation (13,700× *g*, 30 min, 4 °C). We determined the protein concentrations of the resulting supernatants via a BCA assay kit (Beyotime, Shanghai, China). Equal protein amounts (16 μg of total protein in 8 μL per lane) alongside 4 μL of pre-stained protein marker (Thermo Scientific, Waltham, MA, USA, 26619) were resolved by SDS-PAGE and subsequently partitioned onto PVDF membranes (Millipore, Burlington, MA, USA). Following a 10 min blocking step in TBST containing 5% skim milk at room temperature, the membranes were probed overnight at 4 °C with primary antibodies targeting LXR, LDL-R, β-actin (Proteintech, Wuhan, Hubei, China), and ABCA1 (Invitrogen, Carlsbad, CA, USA). After three successive washes with TBST, the membranes were incubated for 1 h with HRP-conjugated secondary antibodies. The protein signals were visualized, and the resulting bands were quantified through grayscale analysis using ImageJ software (version 1.53t). β-actin functioned as the internal loading control for normalization. Comprehensive antibody specifications are detailed in Table S4.

### 2.8. Determination of Bile Acid (BA) Concentration

The feces of pigs were collected and stored at −80 °C, and then the samples were sent to MetWare Biotechnology Co., Ltd. (Wuhan, China), for BA concentration analysis. Detailed methods and BA standard information are displayed in the Supporting Materials. The 65 bile acids are shown in Table S5. Briefly, 20 mg of the feces sample of Bama pigs was weighed and placed in a 2 mL EP tube with 10 μL of internal standard mixed working solution (a concentration of 1 μg/mL), 200 μL of 20% methanol acetonitrile and two steel balls, and centrifuged at 600× *g* and 4 °C for 10 min. The shaken sample was placed in a −20 °C freezer for 10 min. It was re-centrifuged at 13,700× *g* for 10 min. After centrifugation, the supernatant was taken and we waited for LC-MS/MS machine. Finally, BA contents were detected by MetWare (http://www.metware.cn/) based on the AB Sciex QTRAP 6500 liquid chromatography−tandem mass spectrometry (LC-MS/MS) platform.

### 2.9. Fecal Microbiota 16S rDNA Analysis

DNA extraction and library preparation: Fecal specimens were harvested and preserved at −80 °C prior to being dispatched to Metware Biotechnology (Wuhan, China) for sequencing. We extracted the total genomic DNA using the CTAB method [16], with its concentration and integrity verified via 1% agarose gel electrophoresis. The isolated DNA was subsequently adjusted to a working concentration of 1 1 ng/μL using sterile water. To amplify the V3–V4 region of the 16S rDNA, PCR was conducted using specific barcoded primers and Phusion^®^ High-Fidelity PCR Master Mix (New England Biolabs, Ipswich, MA, USA). Sequencing libraries were then generated through the TruSeq^®^ DNA PCR-Free Sample Preparation Kit (Illumina, San Diego, CA, USA).

Quality control and bioinformatic processing: The resulting libraries underwent quality assessment using an Agilent Bioanalyzer 2100 system and a Qubit@ 2.0 Fluorometer. Sequencing was performed on the Illumina NovaSeq platform, generating 250 bp paired-end reads. For downstream bioinformatic analysis, we utilized Uparse software (v7.0.1001) to process the sequences. Individual reads with ≥97% similarity were clustered into the same operational taxonomic units (OTUs). Finally, representative sequences from each OTU were selected for taxonomic annotation.

### 2.10. Short-Chain Fatty Acid (SCFA) Profile Analysis

Similarly, we sent a sample of pig feces stored in −80 °C refrigerator to Metware Biotechnology (Wuhan, China) for SCFAs profile analysis. Detailed methods are displayed in the Supporting Materials. In short, 20 mg of fecal sample was accurately weighed and placed in a 2 mL EP tube. Next, 1 mL of phosphoric acid (0.5% *v*/*v*) solution and a small steel ball were added to the EP tube. The samples were ground uniformly, then vortexed for 10 min and ultrasonicated for 5 min. Then, 100 μL of supernatant was moved into a 1.5 mL centrifugal tube after the mixture was centrifuged at a speed of 12,000 r/min for 10 min at 4 °C. Following this, 500 μL of MTBE (containing internal standard) solution was added to the centrifugal tube and the mixture was vortexed for 3 min followed by ultrasonicating for 5 min. After that, the mixture was centrifuged at a speed of 12,000 r/min for 10 min at 4 °C. The supernatant was collected and used for gas chromatography–mass spectrometry (GC-MS/MS) analysis.

### 2.11. Statistical Analysis

All data are expressed as the mean ± standard error of the mean (SEM). Statistical analyses were performed using GraphPad Prism 9.0 software (GraphPad Software, Inc., La Jolla, CA, USA). Prior to parametric analysis, the normality of data distribution was assessed using the Shapiro–Wilk test, and the homogeneity of variance was confirmed via Levene’s test. Differences among multiple experimental groups were analyzed using one-way analysis of variance (ANOVA), followed by Dunnett’s multiple comparisons post hoc test to determine specific differences between the treatment groups and the Model control group. A value of *p* < 0.05 was considered to indicate a statistically significant difference. For the high-dimensional microbiome and metabolomic correlation analyses, Pearson’s correlation coefficients were calculated using the Metware Cloud platform, with stringent screening criteria (|R| > 0.8 and *p* < 0.05) applied to evaluate correlations and account for potential false positives inherent in multiple comparisons. Statistical significance levels were denoted as follows: * *p* < 0.05, ** *p* < 0.01, *** *p* < 0.001, and **** *p* < 0.0001.

## 3. Results

### 3.1. Effects of COST on Weight and Body Dimension

**Figure 2:**
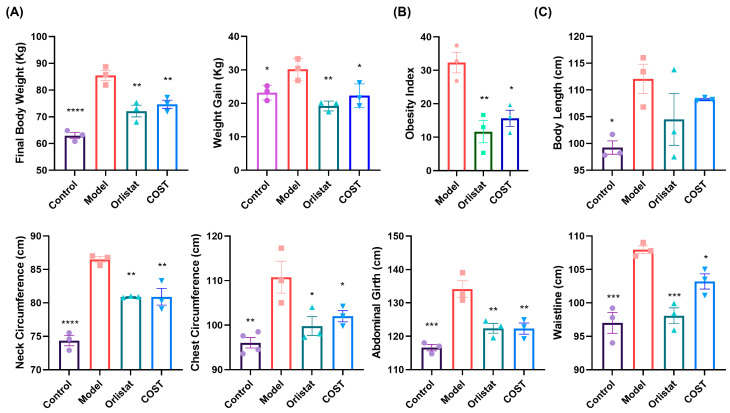
Effects of COST on weight and body dimension. (**A**) Body weight and Weight gain. (**B**) Obesity index. (**C**) Body length, neck circumference, chest circumference, abdominal girth and waistline. Data are expressed as mean ± SEM (*n* = 3–4 per group). Compared with the Model group, * *p* < 0.05, ** *p* < 0.01, *** *p* < 0.001 and **** *p* < 0.0001. The obesity index was calculated as [(actual weight of the treatment group—mean weight of the Control group)/mean weight of the Control group] × 100%, as detailed in Section 2.3.

To evaluate the anti-obesity effects of COST, we monitored body weight and related parameters (detailed data are provided in Table S6). At the beginning of the experiment, there were no significant differences in the initial body weights among the four groups (*p* > 0.05), confirming a consistent baseline before treatments. Pigs in the Model group exhibited a significant increase in body weight compared with the Control group, confirming that HFD feeding successfully induced obesity. In contrast, COST supplementation significantly attenuated this weight gain, with the COST group showing a slower growth trajectory (Figure 2A). Consistently, the obesity index was markedly reduced in COST-treated pigs relative to the Model group (Figure 2B). Weekly food intake was monitored during the 12-week treatment period (weeks 12–24) and is presented in Supplementary Figure S1A. No significant differences in food intake were observed among the Control, Model, Orlistat, and COST groups throughout the treatment period (*p* > 0.05). Feed efficiency (calculated as body weight gain divided by cumulative food intake during the treatment period) was significantly lower in the Orlistat (*p* < 0.01) and COST (*p* < 0.05) groups compared with the Model group (Figure S1B). These results indicate that COST ameliorates HFD-induced obesity without altering food intake but by reducing feed efficiency.

We further assessed body dimensions to evaluate the impact on adiposity distribution. While body length did not differ significantly between HFD-fed groups (Control vs. Model/COST), suggesting that COST does not impair normal skeletal growth, it significantly reduced multiple measures of adiposity. Specifically, COST treatment decreased neck circumference, chest circumference, abdominal circumference, and hip circumference compared with the Model group (Figure 2C). Visceral index data are presented in Figure S2. Collectively, these results demonstrate that HFD feeding successfully induces obesity in Bama mini-pigs, and that COST intervention significantly ameliorates HFD-induced obesity without affecting normal body growth.

### 3.2. Effects of COST on Serum Biochemical Indices

**Figure 3:**
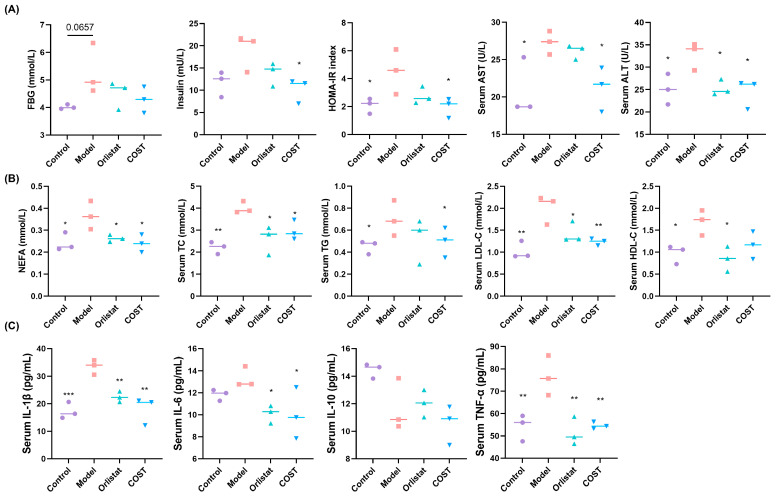
Effects of COST on biochemical indicators in blood. (**A**) Effects of COST on blood glucose indicators, AST and ALT. (**B**) Serum lipids: NEFA, TG, TC, LDL-C and HDL-C. (**C**) Serum IL-6, IL-1β, IL-10 and TNF-α. Data are expressed as mean ± SEM (*n* = 3 per group); the horizontal line in the graph represents the mean. Compared with the Model group, * *p* < 0.05, ** *p* < 0.01 and *** *p* < 0.001.

We next evaluated the impact of COST on metabolic parameters associated with obesity. FBG levels did not differ significantly among groups, indicating that the minipigs had not yet developed overt hyperglycemia. This finding may be attributed to insufficient HFD feeding duration, as suggested by previous studies [17]. However, the elevated HOMA-IR index, driven by compensatory hyperinsulinemia, provided robust evidence of insulin resistance (IR) in the Model group. In contrast, INS was more responsive to the intervention. COST treatment significantly reduced serum INS levels compared with the Model group, leading to a marked decrease in the homeostatic model assessment of HOMA-IR (Figure 3A). Regarding liver function, serum levels of AST and ALT were significantly elevated in the Model group relative to Controls, indicating HFD-induced hepatic injury. Notably, COST supplementation reversed this elevation, restoring AST and ALT to near-control levels (Figure 3A). We then examined serum lipid profiles and inflammatory cytokines. Compared with the Control group, the Model group exhibited significantly higher levels of NEFA, TG, TC, LDL-C, and HDL-C, confirming that HFD feeding successfully induced hyperlipidemia. In contrast, COST treatment significantly reduced these lipid parameters relative to the Model group (Figure 3B). Given that obesity is characterized by chronic low-grade inflammation, we also measured serum inflammatory cytokines. COST treatment significantly decreased the pro-inflammatory cytokines IL-1β, IL-6, and TNF-α, while IL-10 levels remained unchanged (Figure 3C). Collectively, these results demonstrate that COST ameliorates multiple metabolic disturbances associated with HFD-induced obesity, including insulin resistance, liver dysfunction, dyslipidemia, and inflammation.

### 3.3. Effects of COST on Tissue Biochemical Indicators

**Figure 4:**
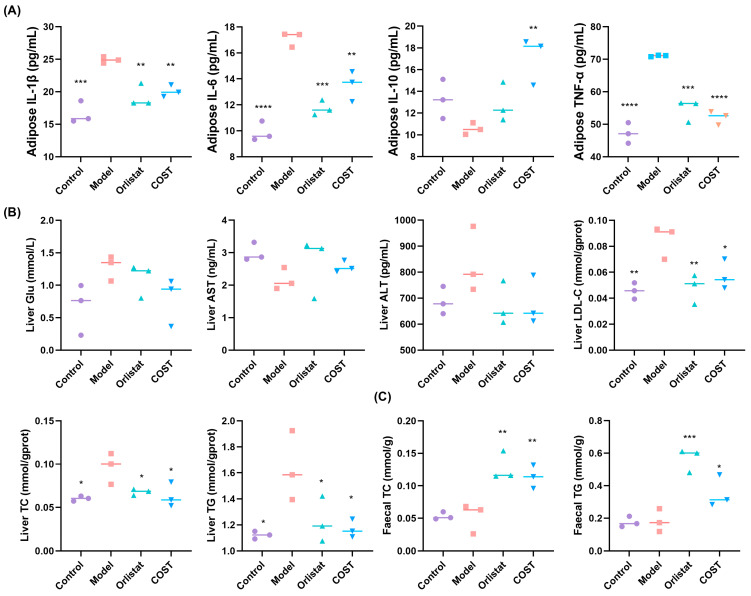
Effects of COST on biochemical indicators in other tissues. (**A**) Adipose tissue IL-1β, IL-6, IL-10 and TNF-α. (**B**) Liver Glu, AST, ALT, LDL-C, TC, and TG. (**C**) Fecal TC and TG. Data are expressed as mean ± SEM (*n* = 3 per group); the horizontal line in the graph represents the mean. Compared with the Model group, * *p* < 0.05, ** *p* < 0.01, *** *p* < 0.001 and **** *p* < 0.0001.

To further elucidate the anti-obesity mechanisms of COST, we examined inflammatory markers in adipose tissue, metabolic parameters in liver tissue, and cholesterol content in feces. In adipose tissue, COST treatment significantly decreased the levels of pro-inflammatory cytokines IL-1β, IL-6, and TNF-α, consistent with the changes observed in serum. Notably, unlike in serum, where IL-10 remained unchanged, adipose tissue IL-10 levels were markedly elevated in the COST group compared with the Model group (Figure 4A), suggesting that COST may promote a local anti-inflammatory environment in adipose tissue.

In liver tissue, Glu, AST, and ALT levels showed no significant differences among groups (Figure 4B). However, consistent with serum lipid profiles, COST treatment significantly reduced hepatic levels of TG, TC, and LDL-C relative to the Model group (Figure 4B).

We next measured fecal lipid content to assess whether COST promotes cholesterol elimination. Compared with the Model group, COST supplementation significantly increased fecal TC and TG levels (Figure 4C). Taken together, these findings demonstrate that COST mitigates obesity and ameliorates key components of metabolic syndrome by suppressing adipose tissue inflammation, reducing hepatic lipid accumulation, and enhancing fecal cholesterol excretion, highlighting its potential for treating obesity-related metabolic disorders.

### 3.4. Effects of COST on Histopathology and the Intestinal Barrier

**Figure 5:**
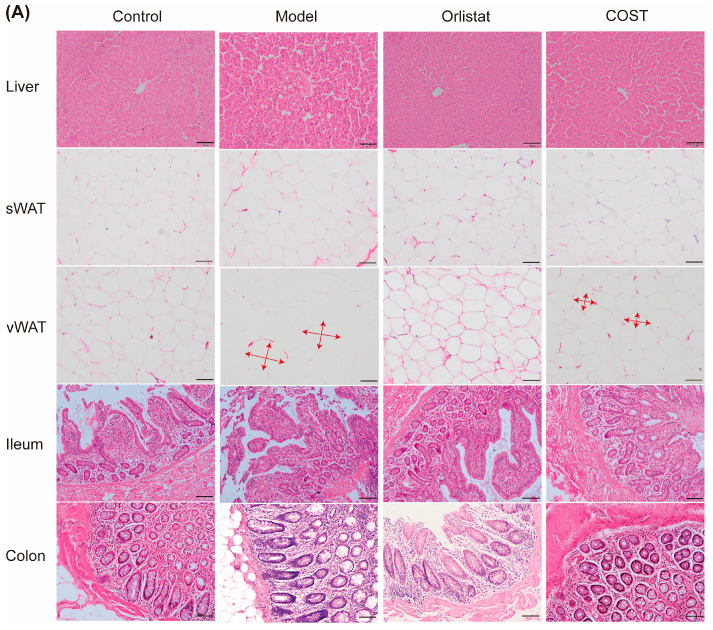

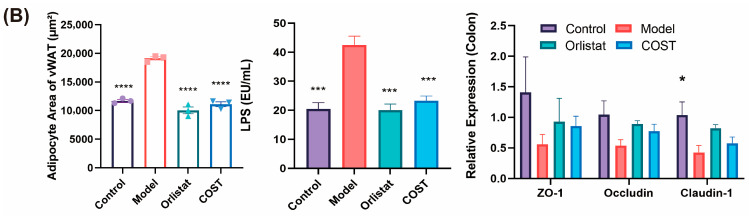
Effects of COST on histopathology of liver, fat and intestine. (**A**) Representative pictures of liver sections (scale bar = 100 μm), white adipose tissue sections (scale bar = 100 μm), ileal sections (scale bar = 100 μm), colon sections (scale bar = 100 μm) stained with H&E. Red arrows indicate fat cell size. (**B**) Semi-quantitative analysis of visceral adipocytes (by image j), serum LPS content and the relative mRNA expression of ZO-1, Occludin, and Claudin-1. Data are expressed as mean ± SEM (*n* = 3 per group). Compared with the Model group, * *p* < 0.05, *** *p* < 0.001 and **** *p* < 0.0001.

The hepatocytes of the Control group were regularly arranged without vesicular degeneration, whereas those of the Model group exhibited extensive macro- and microvesicular steatosis (large and small vacuoles). Following the COST intervention, the fat vacuoles in liver tissue were markedly reduced (Figure 5A). Compared with the Control group, the size of fat droplets in sWAT was slightly increased in the Model group, which was restored to some extent with COST treatment. Meanwhile, the adipocytes of vWAT in the Model group were significantly enlarged with indistinct cell membranes. However, these abnormal changes in vWAT were effectively reversed by the COST intervention; semi-quantitative analysis of visceral adipocytes is shown in Figure 5B.

Numerous studies have shown that HFD can disrupt the integrity of the intestinal barrier, causing large amounts of LPS to enter the blood circulation, which, in turn, activates immune cells (such as monocytes) and triggers inflammation [18]. As indicated by intestinal pathology, the intestinal villi and intestinal muscle layer of the Model group exhibited severe structural damage, and the depth of the intestinal crypt appeared shallower in ileal pathology. Conversely, the gut muscle layers of the Control group and the COST group were relatively intact and the depth of the crypts was better preserved. Furthermore, it was observed that the colon tissue of the Model group showed an obvious inflammatory infiltration and morphological deformity of goblet cells. However, these abnormal changes were largely ameliorated by the COST intervention (Figure 5A). Consistent with the histopathological analysis, serum LPS level was also significantly reduced after COST treatment (Figure 5B). In addition, we performed qPCR experiments to quantify the mRNA expression of colonic tight junction proteins. Consistently, the intervention of COST exhibited a restorative trend, moderately upregulating the mRNA expression of ZO-1, Claudin-1 and Occludin, although the differences did not reach statistical significance when compared to the Model group (Figure 5B). These results suggested COST can attenuate intestinal barrier dysfunction to some extent. Taken together, these results suggest that COST attenuates HFD-induced intestinal barrier dysfunction, likely contributing to its anti-inflammatory effects.

### 3.5. Effects of COST on mRNA and Protein Expressions of Genes Related to Cholesterol Metabolism

**Figure 6:**
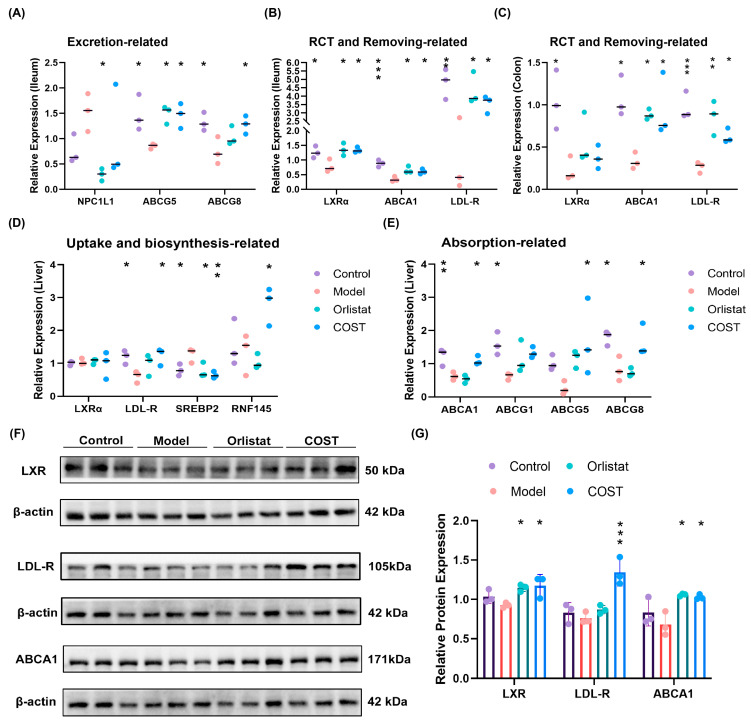
Effects of COST on mRNA and protein expression of genes related to cholesterol metabolism. (**A**) Relative mRNA levels of genes related to cholesterol excretion in the ileum. (**B**) Relative mRNA levels of genes related to RCT and cholesterol clearance in the ileum. (**C**) Relative mRNA levels of genes related to RCT and cholesterol clearance in the colon. (**D**) Relative mRNA levels of genes related to cholesterol uptake and biosynthesis in the liver. (**E**) Relative mRNA levels of genes related to cholesterol absorption in the liver. (**F**) Representative photographs of Western blots. (**G**) The relative expression levels of proteins. For qPCR analysis, the relative mRNA expression levels were normalized to β-actin as the reference gene. Data are expressed as mean ± SEM (*n* = 3 per group); the horizontal line in the graph represents the mean. Compared with the Model group, * *p* < 0.05, ** *p* < 0.01 and *** *p* < 0.001.

To further elucidate the potential underlying mechanisms, we examined the expression of genes involved in cholesterol transport and metabolism. In the intestinal tract, COST treatment significantly upregulated the mRNA expression of *ABCG5* and *ABCG8*, two key transporters mediating cholesterol efflux from intestinal epithelial cells back into the lumen, while the expression of *NPC1L1*, a cholesterol absorption transporter, showed no significant change (Figure 6A). Additionally, COST increased the mRNA levels of *ABCA1* and *LDL-R*, genes involved in RCT and cholesterol uptake, in both ileal and colonic tissues. *LXRα* was significantly upregulated in the ileum but not in the colon (Figure 6B,C). In the liver, COST intervention modulated the expression of genes involved in cholesterol uptake, biosynthesis, and efflux. Specifically, COST significantly increased the mRNA levels of *LDL-R* (cholesterol uptake) and *RNF145* (an LXR target that inhibits cholesterol biosynthesis) while decreasing *SREBP2* (a key activator of cholesterol synthesis) (Figure 6D). Furthermore, hepatic mRNA expression of cholesterol efflux transporters *ABCA1*, *ABCG5*, and *ABCG8* was markedly elevated in the COST group, whereas *ABCG1* showed a modest increase that did not reach statistical significance (Figure 6E; complete gene expression data are provided in Table S7). To confirm these findings at the protein level, we performed WB analysis of key regulators in the liver. Consistent with the mRNA results, COST treatment significantly upregulated the protein expression of LXR, LDL-R, and ABCA1 (Figure 6F,G). Collectively, these results suggest that COST may regulate cholesterol metabolism at both the transcriptional and translational levels, potentially by promoting intestinal cholesterol efflux, enhancing RCT, and modulating hepatic cholesterol uptake and synthesis pathways.

### 3.6. Effects of COST on Fecal Bile Acid Profile

**Figure 7:**
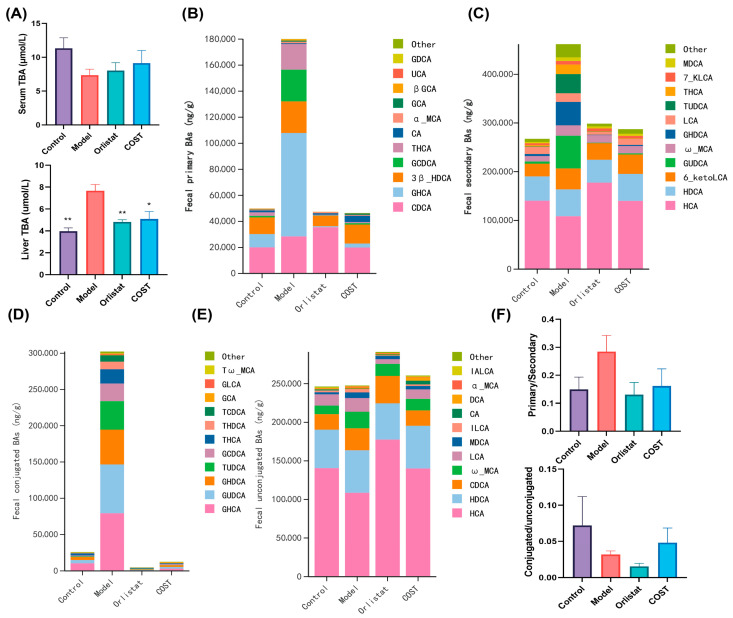
Effects of COST on serum, liver and fecal bile acid profiles. (**A**) Effects of COST treatment on serum TBA (**top**) and liver TBA (**bottom**) levels. (**B**) Fecal primary BAs. (**C**) Fecal secondary BAs. (**D**) Fecal conjugated BAs. (**E**) Fecal unconjugated BAs. (**F**) The ratios of primary to secondary BAs (top) and conjugated to unconjugated BAs (bottom) in feces. Data are expressed as mean ± SEM (*n* = 3–4 per group). BAs profile data is processed via MetWare Cloud Platform (https://cloud.metware.cn/). Compared with the Model group, * *p* < 0.05 and ** *p* < 0.01.

BAs are major end products of cholesterol metabolism and function as key signaling molecules in regulating lipid and glucose homeostasis [19]. We therefore examined BA levels in serum, liver, and feces to assess the impact of COST on BA metabolism. As shown in Figure 7A, serum TBA concentrations showed no significant differences among the experimental groups. In contrast, liver TBA levels were significantly elevated in the Model group compared with the Control group. Notably, COST treatment significantly reduced these hepatic TBA levels, restoring them to levels comparable to the Control’s. Fecal BA composition was further analyzed to assess the impact of COST on BA excretion and modification. Compared with the Model group, COST treatment decreased the fecal levels of primary BAs (Figure 7B), secondary BAs (Figure 7C), and conjugated BAs (Figure 7D), while unconjugated BA content remained largely unchanged (Figure 7E). Consequently, COST intervention visibly reduced the ratio of primary to secondary BAs and increased the ratio of conjugated to unconjugated BAs relative to the Model group (Figure 7F). These findings suggest that COST potentially modulates BA metabolism by altering fecal BA composition and conjugation status.

### 3.7. Effects of COST on Gut Microbiota and SCFAs

**Figure 8:**
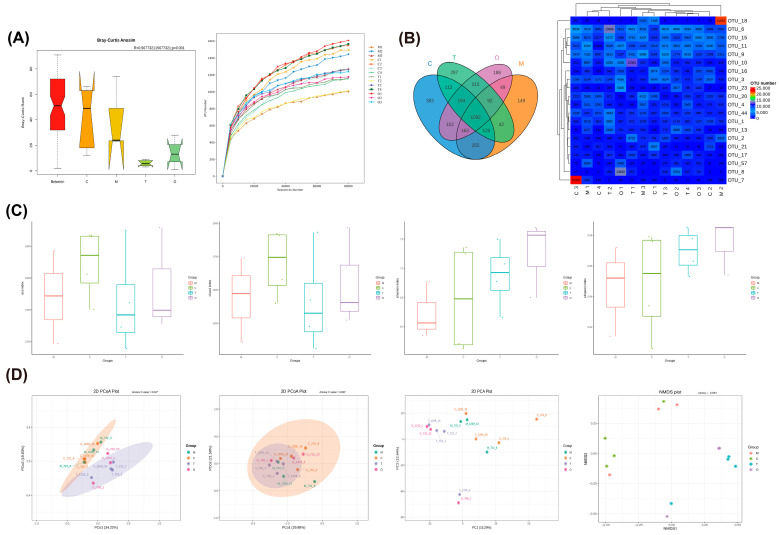
Effects of COST treatment on gut microbiota structure and diversity. (**A**) Analysis of differences in ANOSIM (**left**) and rarefaction curves (**right**) of feces samples. (**B**) Venn diagram (**left**) illustrating the shared and unique OTUs among the different groups, and a heatmap (**right**) displaying the relative abundance of the top 20 OTUs. (**C**) Alpha diversity evaluated by ACE, Chao1, Shannon, and Simpson indices. Red represents the model group; green represents the Control group; blue represents the COST group; purple color represents the Orlistat group. (**D**) Beta diversity analysis including Unweighted UniFrac PCoA, Weighted UniFrac PCoA, PCA, and Non-Metric Multi-Dimensional Scaling (NMDS) plots. Data are expressed as *n* = 3–4 per group. n the panels, C represents the Control group, M represents the Model group, O represents the Orlistat group, and T represents the COST treatment group.

Given that gut microbes metabolize cholesterol-derived BAs and produce SCFAs from dietary polysaccharides [20,21], we performed 16S rDNA sequencing and SCFA profiling on fecal samples to investigate the impact of COST on gut microbial ecology. Sequencing data quality was first evaluated. Analysis of similarities (ANOSIM) based on Bray–Curtis distances revealed a marked difference in microbial community structure among the four groups (R=0.567,P=0.001), and the rarefaction curves approached saturation, indicating sufficient sequencing depth (Figure 8A). Species composition was assessed by clustering effective tags into OTUs at 97% similarity; the shared and unique OTUs among groups, as well as the top 20 OTUs, are presented in Figure 8B.

We then evaluated within-community (alpha) diversity. As shown in Figure 8C, the alpha diversity indices (ACE, Chao1, Shannon, and Simpson) showed no statistically significant differences among the groups (*p* > 0.05), indicating that the overall microbial richness and evenness were not significantly altered by the dietary or pharmacological interventions. However, a stable alpha diversity does not preclude profound changes in microbial community architecture. Between-community (beta) diversity analysis, as depicted in the PCoA and NMDS plots (Figure 8D), revealed significant structural alterations among the groups (AMOVA, *p* < 0.05). While the Model and Control groups exhibited relative overlap in their principal coordinates (particularly evident in the Unweighted UniFrac PCoA), the COST intervention group demonstrated a distinct spatial separation from the Model group. This suggests that the COST intervention significantly shapes the specific taxonomic composition of the gut microbiota, potentially driving structural remodeling rather than a simple quantitative change in species richness. These structural shifts at the community level prompted our subsequent investigation to identify the specific bacterial taxa responsible for these differences at finer taxonomic resolutions.

**Figure 9:**
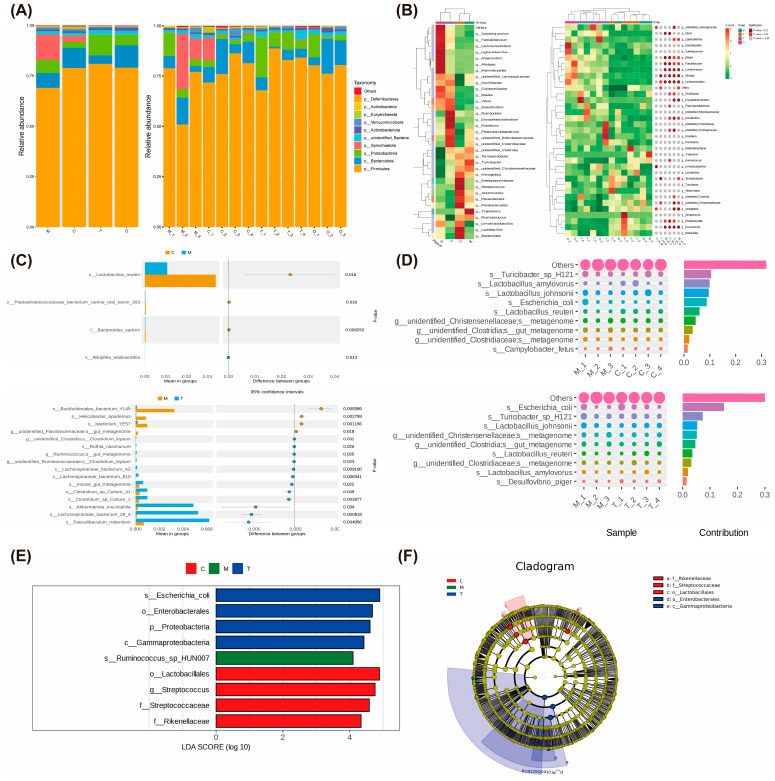
Effects of COST treatment on the species relative abundances. (**A**) Stacked bars of species relative abundance of microbiota at the phylum level. (**B**) Heatmap of species relative abundance of microbiota at the genus level. (**C**) Diagram of species difference analysis on *t*-test at the species level. (**D**) Diagram of SIMPER differential contribution. (**E**) Histogram of LDA value distribution. (**F**) LEfSe analysis evolutionary branch diagram. Data are expressed as *n* = 3–4 per group.

To further characterize these compositional differences, we analyzed the microbial community at multiple taxonomic levels. At the phylum level, *Firmicutes*, *Bacteroidota*, and *Proteobacteria* were the dominant phyla across all groups, collectively accounting for more than 80% of the relative bacterial abundance (Figure 9A). Compared with the Model group, COST treatment resulted in a marked increase in the relative abundances of *Firmicutes* and *Proteobacteria*, accompanied by a decrease in *Bacteroidota* and *Spirochaetota* (Figure 9A). At the genus and species levels, heatmap analyses revealed distinct clustering between groups (Figure 9B). Notably, hierarchical clustering demonstrated that the COST and Orlistat groups clustered together, separating from the Control and Model groups. This pattern suggests that the direct introduction of non-digestible prebiotics (COST) or unabsorbed lipids (due to Orlistat) into the distal gut exerted a more profound structural reshaping pressure on the microbial community than the high-fat diet itself, driving the microbiota into a distinct compositional state.

Specifically, differential analysis based on *t*-tests revealed that COST intervention significantly upregulated specific taxa compared to the Model group, notably including the well-recognized beneficial bacterium *Akkermansia muciniphila*, *Faecalibaculum rodentium*, and several members belonging to the SCFA-producing families *Lachnospiraceae* and *Ruminococcaceae*, as well as *Clostridium* species (Figure 9C). Conversely, COST treatment significantly reduced the abundance of specific taxa, such as the potentially pathogenic *Helicobacter apodemus* and certain members of *Burkholderiales* (Figure 9C). Linear discriminant analysis effect size (LEfSe) was further performed to identify the most characteristic taxa in each group (Figure 9E,F). The Control group was characterized by a high abundance of *Streptococcus*, whereas the COST group was significantly enriched for *Escherichia_coli*, and broader taxa within the *Proteobacteria* phylum as the most discriminative features. Additionally, SIMPER analysis indicated that *Escherichia_coli*, *Turicibacter* sp. H121, and *Lactobacillus_johnsonii* were among the top contributors to the overall dissimilarity between the Control and Model groups (Figure 9D). Collectively, these data suggest that COST intervention markedly reshapes the gut microbiota composition in obese Bama mini-pigs, characterized by phylum-level shifts and specific enrichment or depletion of multiple taxa.

**Figure 10:**
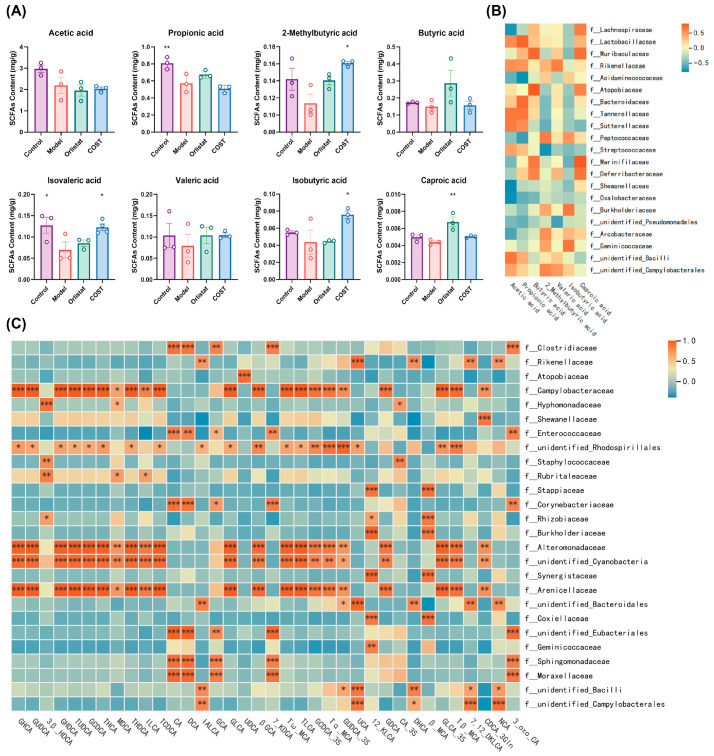
Effects of COST treatment on the intestinal metabolites. (**A**) Contents of SCFAs in colon contents. Data are expressed as mean ± SEM (*n* = 3 per group). (**B**) Correlations between bacterial families and SCFAs. (**C**) Correlations between bacterial families and BAs. Correlation indicators were analyzed by Pearson’s correlation analysis using the Metware Cloud, a free online platform for data analysis (https://cloud.metware.cn). For the heatmaps, statistical significance is indicated by * *p* < 0.05, ** *p* < 0.01, and *** *p* < 0.001. Only bacterial families and BAs/SCFAs with at least one strong correlation (|R| > 0.8) were screened and selected for visualization.

To further explore the potential functional consequences of microbial shifts, we analyzed fecal SCFA levels by GC-MS. The major SCFAs detected in pig feces were classical carbohydrate-derived SCFAs (acetic acid, propionic acid, butyric acid), valeric acid, caproic acid, and the branched-chain SCFAs (BCFAs) including 2-methylbutyric acid, isobutyric acid, and isovaleric acid, which are typically derived from microbial amino acid fermentation. Compared with the Model group, COST supplementation did not significantly alter classical SCFAs but significantly increased the fecal levels of the BCFAs (2-methylbutyric acid, isobutyric acid, and isovaleric acid) (Figure 10A). This specific enrichment indicates a distinct physiological implication, suggesting that COST intervention particularly modulates microbial amino acid fermentation pathways rather than merely classical carbohydrate fermentation.

Correlation analysis was then performed to explore potential associations between the altered microbial taxa and these metabolic changes. As shown in Figure 10B, several families enriched by COST, including *Arcobacteraceae*, *Peptococcaceae*, and *Rikenellaceae*, were positively correlated with the elevated BCFAs (2-methylbutyric acid, isobutyric acid) and other SCFAs (valeric acid, caproic acid). Additionally, specific bacterial families exhibited strong positive correlations with multiple BA species: *Campylobacteraceae*, *Alteromonadaceae*, an unidentified family within *Cyanobacteria*, and *Arenicellaceae* were associated with a wide range of BAs, including GHCA, GUDCA, TCDCA, and their sulfated derivatives (Figure 10C).

## 4. Discussion

The surge in seafood consumption driven by modern diets and lifestyles has led to growing concern regarding the disposal of marine wastes such as shrimp and crab shells [22,23]. Interestingly, these shells have been used in TCM for their properties in resolving stagnation, hinting at bioactive components. Recent technological advances have enabled the extraction of COST, a natural product derived from these shells, which has been shown to exert anti-inflammatory and lipid-lowering activities in rodents [24]. However, rodents exhibit significant metabolic differences from humans, while pigs are recognized as a more suitable animal model for studying metabolic diseases due to their close similarity to humans in metabolic characteristics [25]. Therefore, building upon previous foundational rodent studies, the primary contribution of this study is the comprehensive evaluation of COST in an HFD-induced obese Bama mini-pig model. By integrating macroscopic metabolic parameters, detailed bile acid profiling, and gut microbiota analyses, we aimed to precisely elucidate the complex, multi-target mechanisms of COST, providing a demonstration for the application of pig models in metabolic disease research.

The results of this study indicated that COST intervention effectively reduced body weight, obesity index, and subcutaneous fat thickness, as well as the circumferences of the neck, chest, abdomen, and buttocks in obese Bama mini-pigs, without affecting body length, suggesting that COST does not inhibit normal growth. Meanwhile, COST improved the levels of serum insulin, blood lipids, and anti-inflammatory factors, reduced the levels of liver injury indicators (AST and ALT), and alleviated hepatic lipid accumulation. Pathological observations revealed that COST decreased hepatic lipid droplets, reduced adipocyte size, and visibly repaired the intestinal mucosal barrier damaged by HFD. This structural restoration was accompanied by a highly significant reduction in serum LPS levels and a restorative trend in the mRNA expression of intestinal tight junction proteins (ZO-1, Claudin-1, and Occludin). Importantly, the beneficial effects of COST extend beyond cholesterol metabolism to encompass multiple components of metabolic syndrome. The significant reduction in HOMA-IR indicates improved insulin sensitivity, addressing the insulin resistance component. The normalization of serum and hepatic lipid profiles (TG, TC, LDL-C) directly targets the dyslipidemia component. The anti-inflammatory effects, evidenced by decreased pro-inflammatory cytokines in both serum and adipose tissue, further contribute to ameliorating the chronic low-grade inflammation characteristic of metabolic syndrome. Additionally, the visible restoration of intestinal barrier integrity and the dramatic reduction in serum LPS levels suggest potential benefits for metabolic endotoxemia, an emerging contributor to metabolic dysfunction. Notably, the anti-obesity effects of COST occurred without any alteration in food intake (Supplementary Figure S1A), which remained comparable across all groups, including the Orlistat positive-control group. Furthermore, feed efficiency was significantly reduced in both the Orlistat (*p* < 0.01) and COST (*p* < 0.05) groups compared with the Model group (Supplementary Figure S1B). These findings demonstrate that COST, similar to Orlistat, exerts its beneficial effects primarily through systemic metabolic regulation rather than by suppressing appetite or reducing energy intake. These multifaceted improvements suggest that COST potentially targets the underlying metabolic dysfunction rather than merely reducing caloric intake or absorption, positioning it as a promising dietary bioactive for the comprehensive management of metabolic syndrome.

A core finding of this study is that the anti-obesity effects of COST are closely associated with the regulation of cholesterol metabolism through multiple interconnected pathways, which is consistent with previous rodent studies showing that COS activates LXRs to promote cholesterol conversion to BAs, thereby ameliorating hyperlipidemia [11]. LXRs function as key regulators of cholesterol homeostasis by binding to and modulating the expression of genes involved in cholesterol absorption, transport, efflux, excretion, and conversion to BAs. As the primary gateway for cholesterol entry into the body, the intestine plays a pivotal role in maintaining systemic cholesterol balance [26], and our results highlight the regulatory role of COST in intestinal cholesterol handling.

Dietary cholesterol absorption in the intestinal lumen is primarily mediated by NPC1L1. In our study, COST treatment did not significantly downregulate NPC1L1 expression, suggesting that its cholesterol-lowering effect is not due to inhibited uptake. Instead, we observed a significant upregulation of ABCG5 and ABCG8, transporters responsible for pumping cholesterol back into the intestinal lumen. This suggests that COST promotes transintestinal cholesterol efflux, a mechanism consistent with observations that overexpression of ABCG5/ABCG8 effectively reduces net cholesterol absorption [26]. Additionally, cholesterol efflux from intestinal epithelial cells into the circulatory system is facilitated by the basement membrane protein ABCA1, and COST intervention elevated ABCA1 mRNA levels in the ileum, colon, and liver, further supporting the role of COST in enhancing cholesterol efflux.

Beyond intestinal regulation, COST modulates cholesterol metabolism in the liver, another critical tissue for cholesterol uptake, absorption, and biosynthesis [27,28]. LDL-R, cell surface proteins critical for LDL-C uptake, are responsible for removing more than 80% of circulating LDL-C [29]. Their expression is regulated by SREBP2. Notably, the LXR and SREBP2 pathways act synergistically to maintain sterol balance: when cellular cholesterol levels are elevated, LXRs promote excess cholesterol removal, whereas SREBP2 enhances cholesterol biosynthesis and uptake. SREBP2 can activate LXRs by generating oxysterol-like ligands, thereby upregulating ABCA1 transcription [30]. In our study, COST intervention appeared to tip this balance towards cholesterol clearance: it decreased SREBP2 mRNA (reducing synthesis) while increasing LDL-R mRNA (enhancing uptake). Furthermore, the upregulation of RNF145, a known LXR target gene that inhibits cholesterol biosynthesis, provides additional evidence for LXR activation. These findings indicate that COST modulates the LXR/SREBP2 axis to inhibit hepatic cholesterol synthesis and enhance LDL-C uptake. In addition to regulating cholesterol synthesis and uptake in the liver, COST also modulates reverse cholesterol transport (RCT), another key pathway for cholesterol homeostasis.

RCT is another key pathway by which LXRs maintain cholesterol homeostasis, involving the efflux of excess cholesterol from peripheral tissues (primarily mediated by ABCA1 and ABCG1) to HDL, which then transports it to the liver for conversion to BAs and excretion [31]. ABCA1 mediates the first step of RCT, and LXRs promote RCT to maintain cholesterol balance [26]. Our results showed a significant increase in hepatic ABCA1 mRNA expression in the COST group. WB analysis further validated the PCR results, confirming the upregulation of LXR, LDL-R, and ABCA1 following COST treatment. Collectively, these data suggest that COST reduces systemic cholesterol levels potentially through multiple mechanisms: promoting intestinal cholesterol efflux, enhancing RCT, and inhibiting hepatic cholesterol synthesis and absorption. A simplified mechanistic diagram summarizing these effects is provided in the Supplementary Materials.

In addition to cholesterol metabolism, COST appears to modulate the complex “BAs-gut microbiota” metabolic network, which plays a pivotal role in obesity development [32]. BAs are important end products of cholesterol metabolism, synthesized from cholesterol through a series of hydroxylation and side-chain oxidation steps [33]. Before excretion into bile, BAs are primarily conjugated with taurine or glycine, which is critical for micelle formation, lipid solubilization, and cholesterol homeostasis [34,35]. Conjugated BAs are then deconjugated by gut microbiota and further biotransformed into secondary BAs (e.g., deoxycholic acid [DCA], ursodeoxycholic acid [UDCA], lithocholic acid [LCA]), which are primarily absorbed in the ileum and recycled (95%) back to the liver via enterohepatic circulation [36]. In HFD-induced obese Bama mini-pigs, many BAs were excreted in the feces, but COST reversed this phenomenon, promoting BA reabsorption and maintaining BA homeostasis.

The gut microbiota is a key regulator of energy metabolism, and obesity is associated with microbial dysbiosis characterized by reduced diversity and expansion of sulfate-reducing bacteria and pathogenic species [32]. Such dysbiosis contributes to obesity pathogenesis by influencing fat deposition, intestinal permeability, and chronic low-grade inflammation [7]. Our study found that COST significantly altered gut microbiota composition, specifically upregulating *Firmicutes* and *Proteobacteria*, and downregulating *Spirochaetota* and *Bacteroidota*. Since the COST extract was confirmed to be sterile (Table S1), this microbial shift likely represents a targeted remodeling of the endogenous gut microbiome driven by COST fermentation. Furthermore, we fully agree that interpreting broad taxonomic shifts requires extreme caution. Specifically, the expansion of the *Proteobacteria* phylum, alongside the enrichment of its subordinate genus *Escherichia* in the COST group, presents a complex metabolic scenario. These taxa are frequently characterized as opportunistic pathogens and are traditionally associated with gut dysbiosis. However, they can also bloom in response to specific environmental pressures or prebiotic substrates. In our study, this microbial shift was accompanied by a highly significant reduction in serum LPS levels (*p* < 0.001) and profound histopathological amelioration of the intestinal mucosal barrier. These robust phenotypic readouts suggest that, in this specific porcine model, the expansion of *Proteobacteria* and *Escherichia* did not induce systemic endotoxemia. Instead, their enrichment likely reflects a specialized ecological adaptation driven by the fermentation of COST. Therefore, we cautiously interpret these microbiota changes not as universally ‘beneficial’ shifts, but rather as a profound, context-dependent structural remodeling of the gut microenvironment. The gut microbiota also modifies primary BAs (synthesized by hepatocytes) through deconjugation and dehydroxylation, enhancing BA pool diversity [37].

Additionally, while classical SCFAs (such as acetate, propionate, and butyrate) are major metabolites derived from classical dietary carbohydrate fermentation [38] that broadly promote metabolic health, our study revealed a specific upregulation of 2-methylbutyric acid, isovaleric acid, and isobutyric acid in the COST group. Importantly, these specific metabolites are classified as branched-chain fatty acids (BCFAs), which are primarily derived from the microbial fermentation of branched-chain amino acids. The physiological implications of BCFAs are distinct from those of classical SCFAs. Emerging evidence suggests that BCFAs play unique roles in modulating hepatic lipid metabolism, influencing adipocyte function, and acting as specialized signaling molecules in host energy regulation. This specific enrichment of BCFAs indicates that COST intervention not only alters carbohydrate utilization but also induces a specialized shift towards amino acid fermentation pathways in the gut. Correlation analysis revealed that *Arcobacteraceae*, *Peptococcaceae*, and *Rikenellaceae* were positively correlated with 2-methylbutyric acid, valeric acid, isobutyric acid, and caproic acid, while *Campylobacteraceae*, *Alteromonadaceae*, *Cyanobacteria*, and *Arenicellaceae* were positively correlated with multiple conjugated BAs, including GHCA, GUDCA, GHDCA, and TUDCA. Interestingly, several of these positively associated bacterial families share phylogenetic homology with marine-derived taxa. This unique enrichment pattern is highly plausible given the nature of the intervention. COST, as a marine-derived chitosan oligosaccharide, serves as a specialized prebiotic substrate that selectively drives the expansion of specific microbial populations equipped to ferment marine polysaccharides. Furthermore, while *Campylobacteraceae* is widely recognized as an opportunistic pathogen in the human microbiota, certain species within this family can reside as normal, asymptomatic commensals in the swine intestinal tract. Therefore, the observed correlations in this Bama pig model illustrate a profound synchronous remodeling of the gut microenvironment and metabolite pools driven by COST, rather than implying a direct beneficial function of these specific taxa in humans. Overall, these findings suggest that COST modulates the gut microbiota-BAs-SCFAs network to further support its anti-obesity effects.

Several limitations of the present work necessitate further investigation in subsequent studies. First, regarding mechanistic depth, while our gene expression and multi-omics analyses strongly implicate the LXR pathway and the gut microbiota-BA-SCFA axis, this evidence remains primarily associative. Without direct causal experiments (e.g., targeted LXR inhibition or fecal microbiota transplantation), these pathways should currently be interpreted as potential mechanisms. Second, concerning pathway validation, although we successfully validated core nodes (LXR, LDL-R, ABCA1) at the protein level, the highly complex crosstalk regulating triglyceride excretion and bile acid transport necessitates broader proteomic profiling and comprehensive serum BA monomer profiling to fully delineate this metabolic network. Third, regarding clinical translation, the precise functional impact of enriched marine-associated taxa (such as *Campylobacteraceae*) within the human microbiota requires future humanized-microbiome validation. Furthermore, while rigorous extraction yields non-allergenic chitosan [39,40], strict quality control regarding crustacean allergens [41] remains essential for human application. Finally, concerning experimental scale, expanding the sample size will be crucial to substantiate these findings. Specifically, the limited size of the mixed-sex cohort utilized in this large animal model precluded robust statistical analyses to evaluate potential sex-specific metabolic responses to COST intervention.

## 5. Conclusions

In conclusion, COST exerts protective effects against HFD-induced obesity and metabolic syndrome in Bama pigs by improving multiple metabolic components, including insulin resistance, dyslipidemia, hepatic steatosis, and inflammation. Mechanistically, our findings suggest that COST may activate LXR-mediated pathways to promote cholesterol efflux (via ABCA1, ABCG5/ABCG8), inhibit cholesterol synthesis (via SREBP2 downregulation and RNF145 upregulation), and enhance reverse cholesterol transport. Additionally, COST appears to modulate the gut microbiota-BAs-SCFAs network by reversing microbial dysbiosis, promoting BA enterohepatic circulation, and increasing SCFA production, thereby potentially contributing to the regulation of energy metabolism and intestinal barrier function. This study highlights COST as a promising dietary bioactive compound derived from marine waste for the management of obesity and related metabolic disorders.

## Data Availability

The raw data supporting the conclusions of this article will be made available by the corresponding authors upon reasonable request, without undue reservation.

## Supplementary Materials

The following supporting information can be downloaded at: https://www.mdpi.com/article/10.3390/nu18081233/s1, Figure S1: Weekly food intake and feed efficiency during the 12–week treatment period (weeks 12–24); Figure S2: Effects of COST on visceral index; Figure S3: The official approval document from the Experimental Animal Ethics Committee; Table S1: Certificate of Analysis of the Chitooligosaccharide; Table S2: Dosage administered in each group; Table S3: Primer sequences; Table S4: Antibody information; Table S5: Information on 65 BAs; Table S6: Effects of COST on body weight parameters and obesity index; Table S7: Relative expressions of genes.

## Abbreviations

COST, chitooligosaccharide; HFD, high-fat diet; T2DM, type 2 diabetes mellitus; CVD, cardiovascular disease; TCM, traditional Chinese medicine; FBG, fasting blood-glucose; AST, aspartate aminotransferase; ALT, alanine aminotransferase; TG, triglycerides; TC, total cholesterol; TBA, total bile acid; LDL-C, low-density lipoprotein-cholesterol; HDL-C, high-density lipoprotein-cholesterol; NEFAs, non-esterified fatty acids; INS, insulin; LPS, lipopolysaccharides; TNF-α, tumor necrosis factor α; IL-1β, interleukin 1β; IL-6, interleukin 6; IL-10, Interleukin 10; vWAT, visceral white adipose tissue; sWAT, subcutaneous white adipose tissue; HOMA-IR, insulin resistance index; RT-PCR, real-time PCR; NCBI, national center for biotechnology information database; WB, Western blot; BAs, bile acids; LC-MS/MS, liquid chromatography-tandem mass spectrometry; GC-MS/MS, gas chromatography-mass spectrometry; OTUs, operational taxonomic units; SCFAs, short-chain fatty acids; NPC1L1, Niemann-Pick C1 like protein 1; ABCG5, ATP binding cassette transporters 5; ABCG8, ATP binding cassette transporters 8; RCT, reverse cholesterol transport; LDL-R, low-density lipoprotein receptor; SREBP-2, sterol regulatory element binding transcription factor 2; LXR, liver X receptor; ABCA1, ATP binding cassette subfamily A member 1; RNF145, ring finger protein 145; ABCG1, ATP binding cassette subfamily G member 1; HCA, Hyocholic acid; HDCA, Hyodeoxycholic acid; CA, Cholic acid; GHCA, Glycohyocholic acid; GUDCA, Glycoursodeoxycholic acid; GHDCA, Glycohyodeoxycholic acid; TUDCA, Tauroursodeoxycholic acid; GCDCA, Glycochenodeoxycholic acid; THCA, Taurohyocholic acid; THDCA, Taurohyodeoxycholic Acid (sodium salt); TCDCA, Taurochenodeoxycholic acid; GLCA, Glycolithocholic acid; Tω_MCA, Tauro-ω-muricholic Acid sodium salt; TLCA, taurolithocholic acid; GCDCA_3S, Glycochenodeoxycholic Acid 3 Sulfate Disodium Salt; Tα_MCA, Tauro-α-muricholicAcid sodium salt; GUDCA_3S, Glycoursodeoxycholic Acid 3 Sulfate Sodium; GLCA_3S, glycolithocholic acid-3-sulfate; Tβ_MCA, Tauro-β-muricholic acid.
