# Supplementary material for: Marine-Derived Chitooligosaccharide Attenuates Obesity and Metabolic Syndrome in Bama Pigs Through LXR-Mediated Cholesterol Metabolism and Gut Microbiota Modulation
Source: Nutrients. 2026 Apr 14;18(8):1233. doi: 10.3390/nu18081233 (PMC13118871; doi:10.3390/nu18081233)
Supplement: Supplementary file 1 [file nutrients-18-01233-s001.zip › nutrients-4220387-supplementary.pdf]

Supplementary Information

Specific method for determination of BA concentration

1. Chemicals and reagents

HPLC-grade acetonitrile (ACN) and methanol (MeOH) were purchased from Merck (Darmstadt, Germany). MilliQ water (Millipore, Bradford, USA) was used in all experiments. All of the standards were purchased from CNW (Shanghai, China) and IsoReag (Shanghai, China). Acetic acid and ammonium acetate were bought from Sigma-Aldrich (St. Louis, MO, USA). The stock solutions of standards were prepared at the concentration of 1 mg/mL in MeOH. All stock solutions were stored at -20°C. The stock solutions were diluted with MeOH to working solutions before analysis.

2. Sample preparation and extraction

Samples (20 mg) were extracted with 200 μL methanol/acetonitrile(v/v=2:8) after the samples grinded with ball mill. 10 μL internal standard mixed solution (1 μg/mL) was added into the extract as internal standards (IS) for the quantication. Put the samples at -20°C for 10 min to precipitated protein. Then centrifugation for 10 min (12000 r/min, and 4°C), the supernatant was transferred to the sample bottle for further LC-MS analysis.

3. HPLC Conditions

The sample extracts were analyzed using an LC-ESI-MS/MS system (UHPLC，ExionLC™ AD, https://sciex.com.cn/ ; MS, Applied Biosystems 6500 Triple Quadrupole, https://sciex.com.cn/). The analytical conditions were as follows, HPLC: column, Waters ACQUITY UPLC HSS T3 C18 (100 mm×2.1 mm i.d.， 1.8 μm); solvent system, water with 0.01% acetic acid and 5mmol/L ammonium acetate (A), acetonitrile with 0.01% acetic acid (B); The gradient was optimized at 5% to 40%B in 0.5 min, then increased to 50% B in 4 min, then increased to 75% B in 3 min, and then 75% to 95% in 2.5min, washed with 95%B for 2 min ,finaly ramped back to 5% B (12-14 min); flow rate, 0.35 mL/min; temperature, 40°C; injection volume: 1 μL. The effluent was alternatively connected to an ESI-triple quadrupole-linear ion trap (QTRAP)-MS.

4. ESI-MS/MS Conditions

Linear ion trap (LIT) and triple quadrupole (QQQ) scans were acquired on a triple quadrupole-linear ion trap mass spectrometer (QTRAP), QTRAP® 6500+ LC-MS/MS System, equipped with an ESI Turbo Ion-Spray interface, operating in negative ion mode and controlled by Analyst 1.6.3 software (Sciex). The ESI source operation parameters were as follows: ion source, ESI-; source temperature 550 °C; ion spray

voltage (IS) -4500 V; curtain gas (CUR) was set at 35 psi, respectively. Bile acids were analyzed using scheduled multiple reaction monitoring (MRM). Data acquisitions were performed using Analyst 1.6.3 software (Sciex). Multiquant 3.0.3 software (Sciex) was used to quantify all metabolites. Mass spectrometer parameters including the declustering potentials (DP) and collision energies (CE) for individual MRM transitions were done with further DP and CE optimization. A specific set of MRM transitions were monitored for each period according to the metabolites eluted within this period.

5. Detection of bile acids

Bile acids contents were detected by MetWare (http://www.metware.cn/) based on the AB Sciex QTRAP 6500 LC-MS/MS platform.

Specific method for SCFA profile analysis

1. Chemicals and reagents

Methyl tert-butyl ether (MTBE) was purchased from CNW (CNW Technologies, Germany). MilliQ water (Millipore, Bradford, USA) was used in all experiments. All of the standards were purchased from CNW (Beijing) or aladdin (Shanghai). The stock solutions of standards were prepared at the concentration of 1 mg/mL in MTBE. All stock solutions were stored at -20 °C. The stock solutions were diluted with MTBE to working solutions before analysis.

2. Sample preparation and extraction

20 mg of fecal sample were accurately weighed and placed in a 2 mL EP tube. 1 mL of phosphoric acid (0.5% v/v) solution and a small steel ball were added to the EP tube. The samples were ground uniformly, then vortexed for 10 min and ultrasonicated for 5 min. 100 μL of supernatant was moved into 1.5 mL centrifugal tube after the mixture was centrifuged with a speed of 12000 r/min for 10 min at 4°C. 500 μL of MTBE (containing internal standard) solution was added to the centrifugal tube and the mixture was vortexed for 3 min followed by ultrasonicating for 5 min. After that, the mixture was centrifuged with a speed of 12000 r/min for 10 min at 4°C. The supernatant was collected and used for GC-MS/MS analysis.

3. GC–MS analysis

Agilent 7890B gas chromatograph coupled to a 7000D mass spectrometer with a DB-FFAP column (30 m length × 0.25 mm i.d. × 0.25 μm film thickness, J&W Scientific, USA) was employed for GC-MS/MS analysis of SCFAs. Helium was used as carrier gas, at a flow rate of 1.2 mL/min. Injection was made in the split mode with a split ratio

5:1 and the injection volume was 1 μL. The oven temperature was held at 50°C for 1 min, raised to 220°C at a rate of 18°C/min and held for 5 min. All samples were analyzed in multiple reaction monitoring mode. The injector inlet and transfer line temperature were 250 °C and 230 °C, respectively.

4. Detection of SCFAs

SCFAs contents detected by MetWare (http://www.metware.cn/) based on the Agilent 7890B-7000D GC-MS/MS platform.

Supplemental tables

Table S1. Certificate of Analysis of the Chitooligosaccharide

Item	Standard	Result
Deacetylation (%)	≥85	90.2
Viscosity (cps, 20°C)	<15	<5
M.W (Da)	<3000	1060
Moisture (%)	≤10	5.3
Ash	≤1.0	0.65
Insoluble (%)	≤1.0	<0.1
Pb (mg/kg)	≤2.0	0.16
As (mg/kg)	≤0.5	<0.08
Total plate count	<1000 cfu /g	<100
Colibacillus (cfu /100g)	Negative	<30
Mold and yeast (cfu /g)	<25	<10
Particle size (mesh)	>100 mesh	100 mesh

Table S2. Dosage administered in each group

Groups	Diet	Drugs
Control group	Ordinary diet	-
Model group	High-fat diet	-
Orlistat group	High-fat diet	2.74mg/kg/d
COST group	High-fat diet	250 mg/kg/d

Table S3. Primer sequences

Gene	Forward primer (5'-3')	Reverse primer (5'-3')
β-actin	CCTGTACGCCAACACAGTGC	ATACTCCTGCTTGCTGATCC
LXRα	CAGTCATACCGGGGACCCT	ATTACCAAGGGACTGTCCTGA
SREBP2	GGGACATCGACGAGATGCTG	TGCTCTGAGAACAAGTCGGG
RNF145	GCTGCAGCACTCCTTACTCA	CCTTCTGTCATGCCCCGATT
ABCA1	TGCCTCCTCCACAGAGAAAAC	AGGTCTTCACCAGGTAATCCG
ABCG1	GGCAGGAGAGAGATGGTCAAG	AGGATTGTTGACGAGCTCCAG
ABCG5	GGGGAGCTCCAAGTACACAG	GGTCCCACGTGATAGCTGAC
ABCG8	GTGTCCTTCCTCCGATGGTG	TGGCATTGAGGATTGCGTCT
LDL-R	CAAGAGCGCACACAGTGAAC	CCCACATTTCTCTTCCACTGC
NPC1L1	TCTGAACGACATGCCCAACA	GCCATGAACCGAGAGGCTAA
ZO-1	GAAATACCTGACGGTGCTGC	GAGGATGGCGTTACCCACAG
Claudin-1	GAGGATGGGCGAGCAGTC	AGTCTGTGCCAATTGAGGCT
Occludin	CAGGTGCACCCTCCAGATTG	TGGACTTTCAAGAGGCCTGG

Table S4. Antibody information

Antibodies	Manufacturer	Catalog No.	Dilution
β-actin	Protein tech	20536-1-AP	1:2000
LXR-a	Protein tech	14351-1-AP	1:2000
ABCA1	Invitrogen	PA1-16789	1:500
LDL-R	Protein tech	10785-1-AP	1:1000

Table S5. Information on 65 BAs

Number	BAs	abbreviation
1	Taurolithocholic acid-3-sulfate	TLCA-3S
2	Dehydrolithocholic acid	DLCA
3	Isoallolithocholic acid	IALCA
4	Isolithocholic acid	ILCA
5	Lithocholic acid	LCA
6	5α-CHOLANIC ACID-3α-OL	alloLCA
7	Nor-Deoxycholic Acid	23-DCA

8	5-β-Cholanic Acid-3α-ol-6-one	6-ketoLCA
9	7-ketolithocholic acid	7-KLCA
10	12-ketolithocholic acid	12-KLCA
11	3-oxodeoxycholic acid	3-oxo-DCA
12	Murideoxycholic acid	MDCA
13	3β-Ursodeoxycholic Acid	3β-UDCA
14	β-Hyodeoxycholic Acid	3β-HDCA
15	Ursodeoxycholic acid	UDCA
16	Hyodeoxycholic acid	HDCA
17	Isochenodeoxycholic Acid	isoCDCA
18	3β-deoxycholic acid	3β-DCA
19	Chenodeoxycholic acid	CDCA
20	Deoxycholic acid	DCA
21	Isodeoxycholic acid	IDCA
22	Norcholic acid	NCA
23	Dehydrocholic acid	DHCA
24	7,12-diketolithocholic acid	7,12-DKLCA
25	6,7-diketolithocholic acid	6,7-DKLCA
26	7-Ketodeoxycholic acid	7-KDCA
27	12-Oxochenodeoxycholic acid	12-oxo-CDCA
28	3-Oxocholic acid	3-oxo-CA
29	Ursocholic acid	UCA
30	ω-muricholic acid	ω-MCA
31	3β-Cholic Acid	3β-CA
32	α-muricholic acid	α-MCA
33	β-muricholic acid	β-MCA
34	Hyocholic acid	HCA
35	Cholic acid	CA
36	Glycolithocholic acid	GLCA
37	Glycoursodeoxycholic acid	GUDCA
38	Glycochenodeoxycholic acid	GCDCA
39	Glycodeoxycholic acid	GDCA
40	Lithocholic acid-3-sulfate	LCA-3S
41	Glycodehydrocholic acid	GDHCA
42	3β-Glycocholic Acid	βGCA
43	Glycohyocholic acid	GHCA

44	Glycocholic acid	GCA
45	Taurolithocholic acid	TLCA
46	Tauroursodeoxycholic acid	TUDCA
47	Taurochenodeoxycholic acid	TCDCA
48	Taurodeoxycholic acid	TDCA
49	Taurodehydrocholic acid	TDHCA
50	Glycolithocholic acid-3-sulfate	GLCA-3S
51	Tauro-β-muricholic acid	Tβ-MCA
52	Tauro-ω-muricholic Acid sodium salt	Tω-MCA
53	Tauro-α-muricholicAcid sodium salt	Tα-MCA
54	Taurohyocholic acid	THCA
55	Taurocholic acid	TCA
56	Chenodeoxycholic acid-3-β-D-glucuronide	CDCA-3Gln
57	Glycohyodeoxycholic Acid	GHDCA
58	Taurohyodeoxycholic Acid (sodium salt)	THDCA
59	Cholic acid 7 sulfate	CA-7S
60	Cholic Acid 3 Sulfate Sodium Salt	CA-3S
61	Chenodeoxycholic acid3-sulfate disodium salt	CDCA-3S
62	Deoxycholic Acid 3-O-Sulfate Disodium Salt	DCA-3-O-S
63	Glycoursodeoxycholic Acid 3 Sulfate Sodium	GUDCA-3S
64	Glycochenodeoxycholic Acid 3 Sulfate Disodium Salt	GCDCA-3S
65	Taurocholic Acid 3 sulfate sodium salt	TCA-3S

Table S6. Effects of COST on body weight parameters and obesity index

Groups	Control	Model	Orlistat	COST
Initial weight (Kg)	25.03±0.7860	27.83±0.9280	27.80±1.060	27.30±1.044
Weight (Kg)	62.93±1.16****	85.43±1.97	72.10±2.16**	74.67±1.53**
Weight gain (Kg)	23.20±1.29*	30.17±1.89	19.20±0.85**	22.30±2.02*
Obesity index	\	32.30±3.07	11.63±3.33**	15.63±2.40*

Footnotes: Data are expressed as mean ± SEM (n = 3 per group). * p< 0.05, **p < 0.01, ***p < 0.001 and ****p < 0.0001 compared with the Model group.

Table S7. Relative expressions of genes

Gene	Control	Model	Orlistat	COST
**Ileum**				
**NPC1L1**	0.7640±0.1660	1.528±0.2164	0.2900±0.06946*	1.000±0.5373
**ABCG5**	1.475±0.2056*	0.8517±0.02861	1.490±0.1024*	1.463±0.1419*
**ABCG8**	1.323±0.1039*	0.7467±0.1549	1.038±0.1097	1.277±0.1036*
**LXRα**	1.263±0.1180*	0.7850±0.1279	1.355±0.1238*	1.342±0.05294*
**ABCA1**	0.8850±0.0682***	0.3417±0.04879	0.6573±0.06783*	0.6037±0.04905*
**LDL-R**	4.788±0.5234**	1.082±0.8192	4.348±0.5593*	3.549±0.3045*
**Colon**				
**LXRα**	1.040±0.2032*	0.2310±0.08277	0.5657±0.1738	0.3753±0.08072
**ABCA1**	1.075±0.1411*	0.3320±0.05710	0.8840±0.03573*	0.9490±0.2184*
**LDL-R**	0.9733±0.09491***	0.2633±0.03768	0.8567±0.1183**	0.6260±0.05027*
**Liver**				
**LXRα**	1.001±0.03642	1.041±0.05505	1.057±0.04584	0.9727±0.2386
**LDL-R**	1.196±0.1210*	0.6013±0.1033	0.9767±0.1880	1.227±0.1523*
**SREBP2**	0.7947±0.1011*	1.263±0.1271	0.7723±0.1289*	0.6450±0.05840**
**RNF145**	1.554±0.4116	1.334±0.3611	1.037±0.1300	2.787±0.3352*
**ABCA1**	1.221±0.1510**	0.6257±0.06801	0.5377±0.06912	1.087±0.07878*
**ABCG1**	1.589±0.2004*	0.6240±0.05622	1.155±0.2842	1.330±0.09522
**ABCG5**	1.016±0.1308	0.2577±0.1189	1.159±0.1516	1.707±0.6664*
**ABCG8**	1.787±0.1257*	0.8277±0.2128	0.7370±0.07114	1.657±0.2830*

Footnotes: Data are expressed as mean ± SEM (n = 3 per group). * p< 0.05, **p < 0.01 and ***p < 0.001 compared with the Model group. Abbreviations: NPC1L1, Niemann-Pick C1 like protein 1; ABCG5, ATP binding cassette transporters 5; ABCG8, ATP binding cassette transporters 8; LXRa, liver X receptor alpha; ABCA1, ATP binding cassette subfamily A member 1; LDL-R, low-density lipoprotein receptor; SREBP2, sterol regulatory element-binding protein 2; RNF145, ring finger protein 145; ABCG1, ATP binding cassette subfamily G member 1.

Supplemental figures

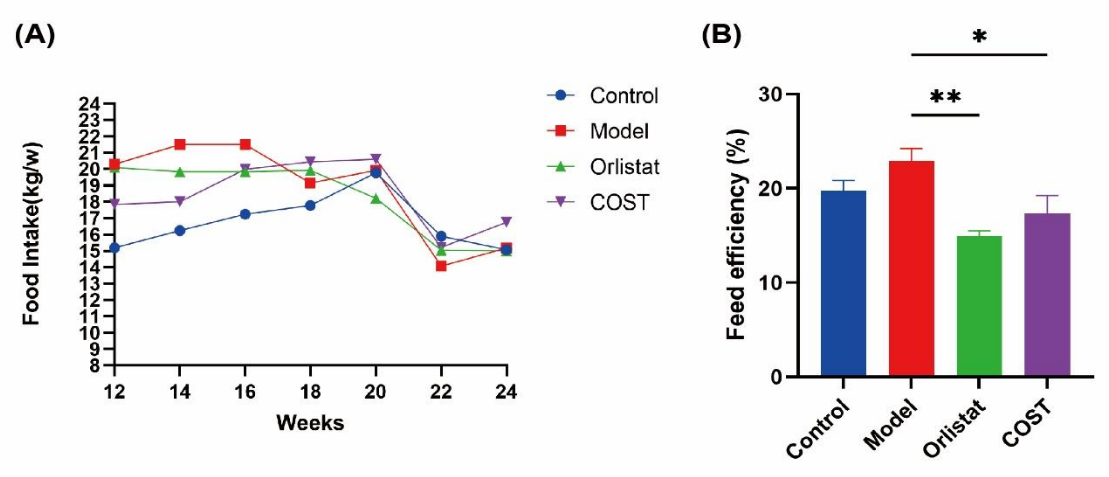

Figure S1. Weekly food intake and feed efficiency during the 12-week treatment period (weeks 12–24). (A) Weekly food intake (kg/w). Data are presented as mean values. No significant differences were observed among the Control, Model, Orlistat, and COST groups ($p > 0.05$, two-way ANOVA). (B) Feed efficiency (%) during the treatment period, calculated as (body weight gain during treatment / cumulative food intake during treatment) × 100%. Data are expressed as mean ± SEM (n = 3 per group). *$p < 0.05$, **$p < 0.01$ vs. Model group (one-way ANOVA followed by post-hoc test).

Weekly food intake was monitored during the 12-week treatment period (weeks 12–24) and is presented in Supplementary Figure S1A. No significant differences in food intake were observed among the Control, Model, Orlistat, and COST groups throughout the treatment period (P > 0.05). Feed efficiency (calculated as body weight gain divided by cumulative food intake during the treatment period) was significantly lower in the Orlistat (P < 0.01) and COST (P < 0.05) groups compared with the Model group (Figure S1B). These results indicate that COST ameliorates HFD-induced obesity without altering food intake, but by reducing feed efficiency.

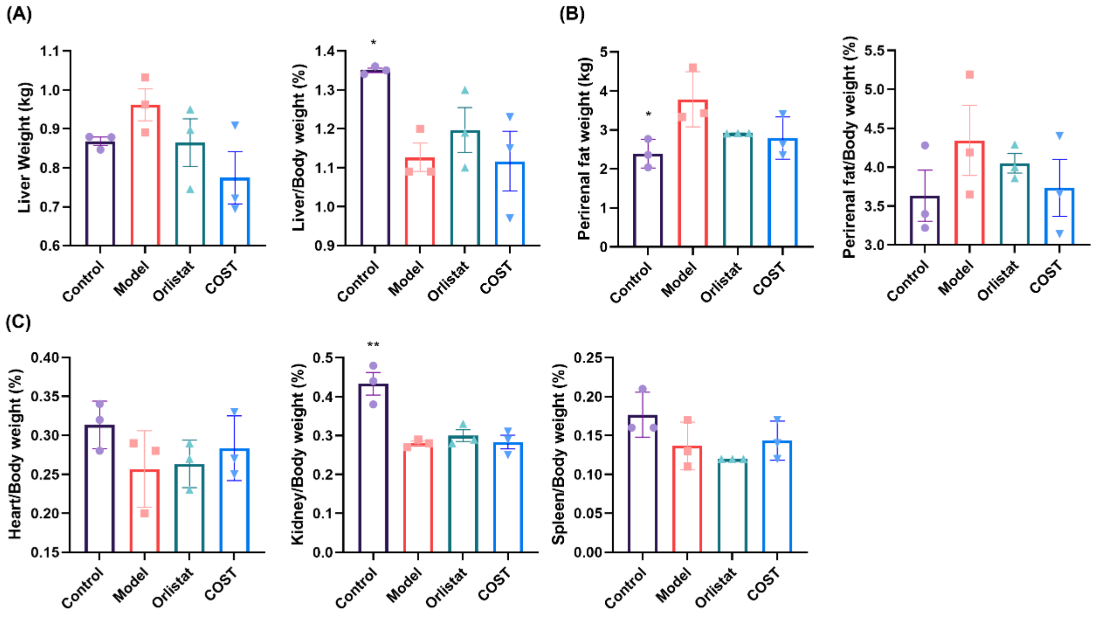

Figure S2. Effects of COST on visceral index. (A) Liver weight and Liver index. (B) perirenal fat weight and perirenal fat index (C). Heart index, Kidney index and Spleen index. Data are presented as "mean ± standard error"; n=3. Compared with the Model group, * $p< 0.05$, ** $p < 0.01$.

The organ index, which is the ratio of the organic weight to body weight, is a commonly indicator in toxicology to determine whether drug samples have toxicological effect on experimental animals. There was no significant difference in the net weight of liver tissue among groups, while the liver index in the Control group was increased compared with other groups (Figure S2A). We consider the reason is related to body weight difference rather than the liver damage, as like renal index in the control group (Figure S2C). Compared with the Model group, the perirenal fat weight in the Control group was significantly reduced, and there was slight decline in the Orlistat group and the COST group without significant change (Figure S2B). The heart index, spleen index and fat index of Bama pigs were not significant among the groups of Bama pigs (Figure S2B, C).

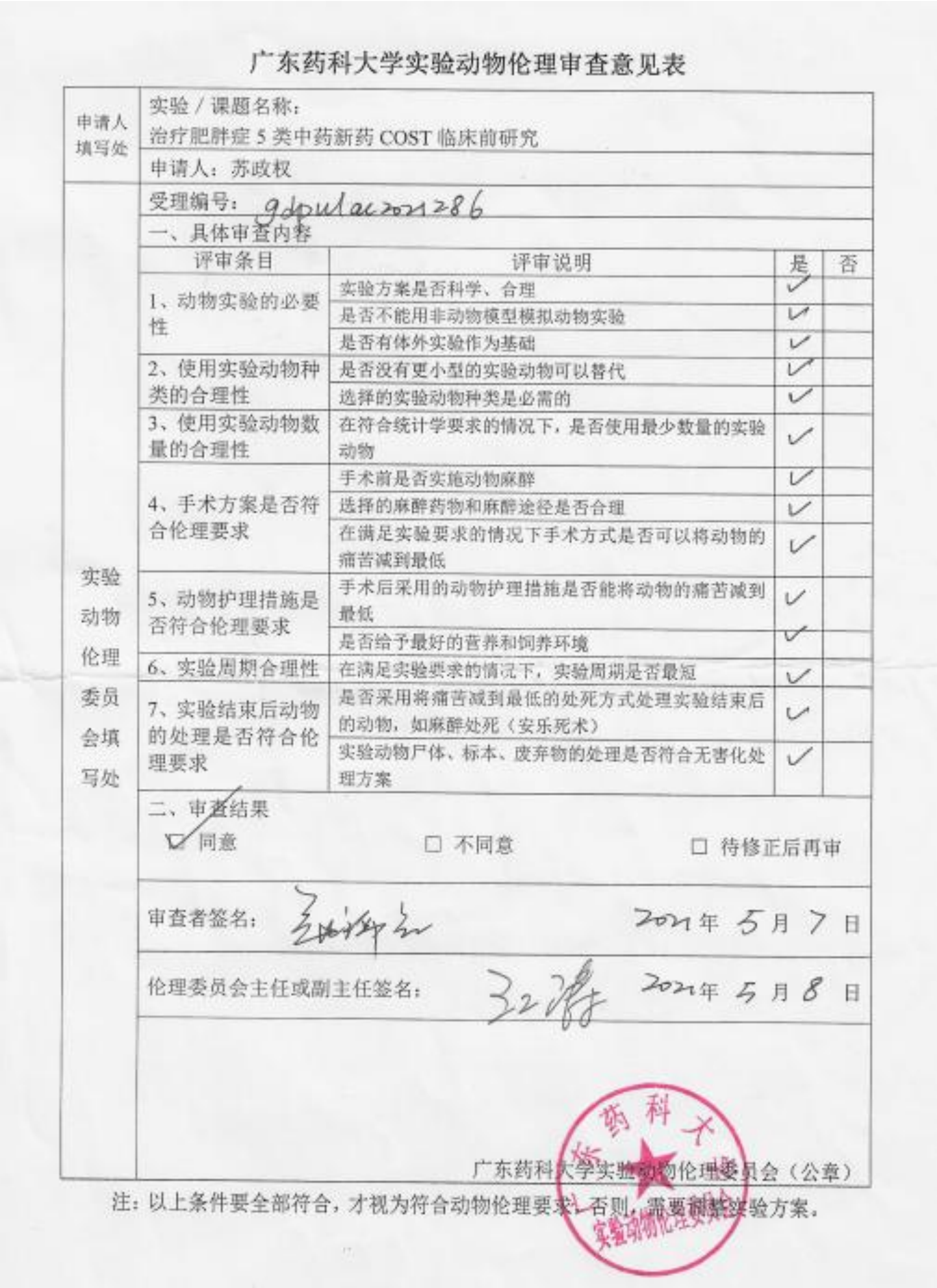

广东药科大学实验动物伦理审查意见表

申请人填写处	实验／课题名称：<br>治疗肥胖症 5 类中药新药 COST 临床前研究			
	申请人：苏政权			
实验动物伦理委员会填写处	受理编号：gdpulac2021286			
	一、具体审查内容			
	评审条目	评审说明	是	否
	1、动物实验的必要性	实验方案是否科学、合理	✓	
		是否不能用非动物模型模拟动物实验	✓	
		是否有体外实验作为基础	✓	
	2、使用实验动物种类的合理性	是否没有更小型的实验动物可以替代	✓	
		选择的实验动物种类是必需的	✓	
	3、使用实验动物数量的合理性	在符合统计学要求的情况下，是否使用最少数量的实验动物	✓	
	4、手术方案是否符合伦理要求	手术前是否实施动物麻醉	✓	
		选择的麻醉药物和麻醉途径是否合理	✓	
		在满足实验要求的情况下手术方式是否可以将动物的痛苦减到最低	✓	
	5、动物护理措施是否符合伦理要求	手术后采用的动物护理措施是否能将动物的痛苦减到最低	✓	
		是否给予最好的营养和饲养环境	✓	
	6、实验周期合理性	在满足实验要求的情况下，实验周期是否最短	✓	
	7、实验结束后动物的处理是否符合伦理要求	是否采用将痛苦减到最低的处死方式处理实验结束后的动物，如麻醉处死（安乐死术）	✓	
		实验动物尸体、标本、废弃物的处理是否符合无害化处理方案	✓	
	二、审查结果<br>☑ 同意　□ 不同意　□ 待修正后再审			
	审查者签名：[signature]　2021 年 5 月 7 日			
	伦理委员会主任或副主任签名：[signature]　2021 年 5 月 8 日			
	广东药科大学实验动物伦理委员会（公章）			

注：以上条件要全部符合，才视为符合动物伦理要求。否则，需要修改实验方案。

Figure S3. The official approval document from the Experimental Animal Ethics Committee. All animal experiments conducted in this preclinical study were formally reviewed and approved by the Experimental Animal Ethics Committee of Guangdong Pharmaceutical University. The official document is provided in Chinese. Key information includes: Institution: Guangdong Pharmaceutical University; Approval Number: gdpulac2021286; Date of Approval: May 8, 2021; Evaluation Result: Approved (Agreed).
